# Microbiome Clusters Disclose Physiologic Variances in Dairy Cows Challenged by Calving and Lipopolysaccharides

**DOI:** 10.1128/mSystems.00856-21

**Published:** 2021-10-19

**Authors:** Johanna Tröscher-Mußotter, Johan S. Saenz, Sandra Grindler, Jennifer Meyer, Susanne U. Kononov, Beate Mezger, Daniel Borda-Molina, Jana Frahm, Sven Dänicke, Amélia Camarinha-Silva, Korinna Huber, Jana Seifert

**Affiliations:** a HoLMiR—Hohenheim Center for Livestock Microbiome Research, University of Hohenheim, Stuttgart, Germany; b Institute of Animal Science, University of Hohenheim, Stuttgart, Germany; c Institute of Animal Nutrition, Friedrich-Loeffler-Institut, Federal Research Institute for Animal Health, Braunschweig, Germany; Teagasc Food Research Centre

**Keywords:** microbiome clusters, dairy cow, metabolome, *Bifidobacterium*, calving, transition, lipopolysaccharide, microbiome clusters

## Abstract

Dairy cows respond individually to stressful situations, even under similar feeding and housing conditions. The phenotypic responsiveness might trace back to their microbiome and its interactions with the host. This long-term study investigated the effects of calving, lipopolysaccharide (LPS)-induced inflammation, and l-carnitine supplementation on fecal bacteria and metabolites, dairy cow milk production, health, energy metabolism, and blood metabolites. Fifty-four multiparous Holstein dairy cows were examined over a defined period of life (168 days). The obtained data allowed a holistic analysis combining microbiome data such as 16S rRNA amplicon sequencing and fecal targeted metabolome (188 metabolites) with host parameters. The conducted analyses allowed the definition of three enterotype-like microbiome clusters in dairy cows which could be linked to the community diversity and dynamics over time. The microbiome clusters were discovered to be treatment independent, governed by *Bifidobacterium* (C-Bifi), unclassified (uncl.) *Clostridiales* (C-Clos), and unclassified *Spirochaetaceae* (C-Spiro). Animals between the clusters varied significantly in terms of illnesses, body weight, microbiome composition, and milk and blood parameters. C-Bifi animals were healthier and leaner with a less diverse but dynamic microbiome. C-Spiro animals were heavier, but the diversity of the static microbiome was higher. This pioneering study uncovered microbiome clusters in dairy cows, each contributing differently to animal health and productive performance and with a crucial role of *Bifidobacterium*.

**IMPORTANCE** The health of dairy cows has to be carefully considered for sustainable and efficient animal production. The microbiome of animals plays an important role in the host’s nutrient supply and regulation of immune functions. We show that a certain composition of the fecal microbiome, called microbiome clusters, can be linked to an animal’s health at challenging life events such as calving and inflammation. Cows with a specific set of bacteria have coped better under these stressors than have others. This novel information has great potential for implementing microbiome clusters as a trait for sustainable breeding strategies.

## INTRODUCTION

For some time, dairy cow breeding has focused on phenotypic ideals with no regard for intestinal bacterial communities, which have evolved within, or for the cow itself across evolution. The intestinal symbionts of ruminants are crucial for proper fiber degradation, fermentation, vitamin production, and host immune functions (1, 2). Even though natural life expectancy can reach up to 20 years, modern dairy cows live between 4.5 and 6 years (3, 4), since production diseases, such as claw lesions and lameness, rumen and hindgut acidosis, ketosis, and reproduction disorders have become more common among herds (5, 6). These diseases often originate from a nonphysiological, high-energy diet necessary for high milk yields; however, it causes perturbation of the established microbiome (1, 6). This diet malnourishes protective, mucus-stimulating gut bacteria, such as *Bifidobacterium* (7), and promotes potentially pathogenic consortia (8). Even though it promotes high productivity, this new microbial community is accompanied by gastrointestinal acidosis, followed by epithelial leakiness (9). The decrease in mucus thickness soon exposes the underlying bare gut epithelia and increases the risk of infection (6, 7, 9). Such a “leaky gut” poses open gates for lipopolysaccharide (LPS) and other immune triggers, reaching the bloodstream and contributing to a latent inflammatory status (6, 8–12). This cascade of increased intestinal stress makes the host more sensitive to infections or health issues, thereby triggering a downward spiral of the physiological state. The latest studies have associated the aforementioned health issues with an impaired gut microbiome (9, 12), suggesting that the modern dairy industry should focus on maintaining gut health, including its complex ecosystem and integrity, to increase the cows’ well-being and performance (9). Additional stressors, such as calving or infection, can pose the final blow for the animal. In particular, calving, and the subsequent transition period, are the most critical and energy-requiring periods in the dairy cow’s life. Energy metabolism in animals is crucial for coping with physiological challenges. However, excessive mobilization of body fat and enhanced ratios of energy in the diet can cause a metabolic imbalance (5). Therefore, finding an optimal balance between the animals’ demands by nature and high performance is a difficult task. Feed supplementation with l-carnitine (CAR), a metabolite inevitably necessary for the transport of long-chain fatty acids into the mitochondria for β-oxidation, is suggested to enhance the energetic potential of dairy cows (13). Cows with an improved energy metabolism could emerge better from stressful phases.

The combined analysis of the fecal microbiome, its metabolites, animal performance variables, and health indicators has the potential to elucidate and understand the cow in its complexity. Hence, the objective of this study was to identify the role of the microbiome in the health of dairy cows during challenging periods. The present work tested physiological and the microbial differences between individuals of the same herd during the calving, transition, and an LPS-induced inflammatory challenge with or without supplementation with rumen-protected l-carnitine.

## RESULTS

**FIG 1:**
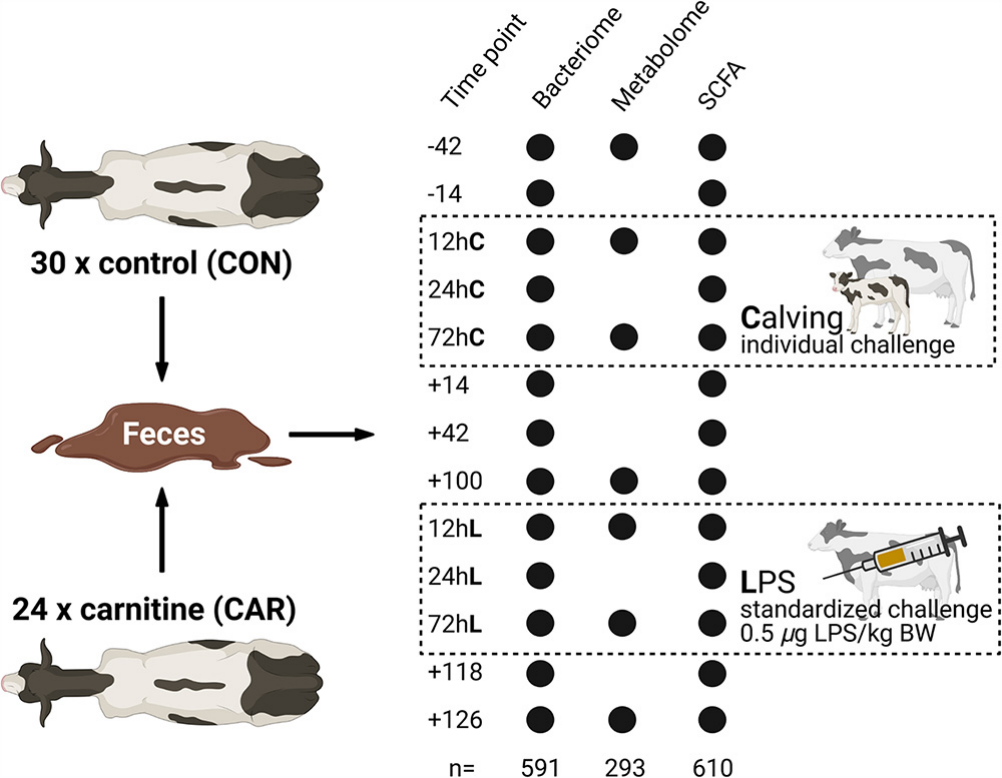
Trial setup of the MitoCow study, including fecal sampling at 13 time points for 16S rRNA sequencing and SCFA measurement as well as at seven time points for Biocrates p180 metabolite analysis. *n* values are the total number of samples measured per analysis. Time points including a “−” or “+” indicate days antepartum or postpartum, respectively, and those including “hC” or “hL” are samples taken at 12, 24 or 72 h after calving or LPS challenge, respectively.

**FIG 2:**
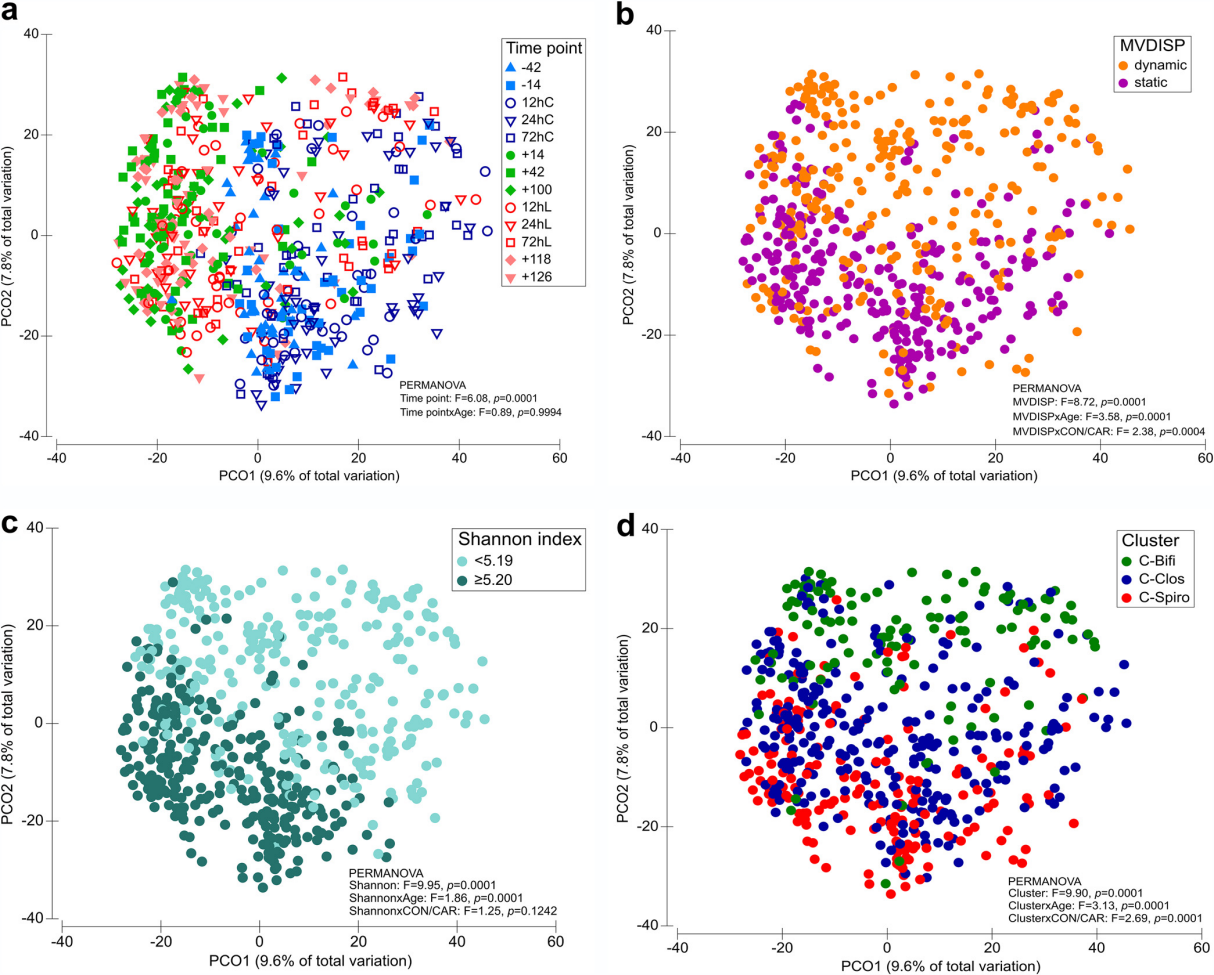
Microbiome dynamics and diversity are associated with defined microbiome clusters. Microbiome analyses included 591 samples of 54 animals with various labels. (a) Time points (time points including a “–” or “+” indicate days antepartum or postpartum, respectively, and time points including “hC” or “hL” are samples taken at 12, 24 or 72 h after calving or LPS challenge, respectively); (b) MVDISP (multivariate dispersion) classification of 26 dynamic and 28 static animals; (c) Shannon diversity indexes separated at the average; (d) animal clusters. PCoA data based on Bray-Curtis metrics showed clustering of operational taxonomic units (OTUs).

The present study investigated 54 multiparous Holstein dairy cows over 168 days, including calving and an inflammatory challenge induced by LPS injection. Fecal samples were collected at 13 time points to obtain a representative overview of the microbiome changes in the host during two physiological challenges (Fig. 1; see Data Sets S1 to S3 at https://github.com/SebasSaenz/Troscher-Mussotter_Cow-enterotypes_2021/tree/main/Datatsets). (Time points including a “−” or “+” indicate days antepartum or postpartum, and those including “hC” or “hL” are samples taken at 12, 24, or 72 h after calving or LPS challenge, respectively.) The herd was randomly split into l-carnitine-supplemented (CAR) and nonsupplemented (CON) cows, defined hereafter as treatment. In general, the time point of sampling had a significant and stronger impact on operational taxonomic unit (OTU)-based community structures (Fig. 2a; analysis of similarity [ANOSIM] global *R* = 0.298, *P = *0.0001; permutational multivariate analysis of variance [PERMANOVA] *F* = 6.08, *P = *0.0001) see Fig. S1 in the supplemental material) than the age of the animal (ANOSIM global *R* = −0.001, *P = *0.504; PERMANOVA *F* = 3.69, *P = *0.0001) or treatment (ANOSIM global *R* = 0.01, *P = *0.006; PERMANOVA *F* = 2.67, *P = *0.0001), with drastic changes after calving and feed adjustment (time point +14). Before calving (time point −14), the microbiomes show the highest within-time similarity (similarity percentage [SIMPER] average at the OTU level = 29.3%), as greater variations among individual animal communities are seen at all other time points (see Data Set S4 at https://github.com/SebasSaenz/Troscher-Mussotter_Cow-enterotypes_2021/tree/main/Datatsets). The highest dissimilarity between time points at the OTU level was observed between 12 h after calving (12hC) and time point +42 (86.7%). The highest genus contribution to time point similarity was observed at 72hC by *Bifidobacterium* (OTU1 and OTU2), accounting for 31.3% of the total similarity.

10.1128/mSystems.00856-21.1 FIG S1 Relative abundance of families (a) and genera (b) in dairy cow fecal samples with an average abundance of ≥0.1% across 13 time points or 168 days. Time points including a “–” or “+” indicate days antepartum or postpartum, respectively, and time points including “hC” or “hL” are samples taken at 12, 24, or 72 h after calving or LPS challenge, respectively. *n* values are the total number of samples averaged per time point. Abbreviation: g.i.s., genera_incertae_sedis. Download FIG S1, TIF file, 0.5 MB. Copyright © 2021 Tröscher-Mußotter et al. 2021 Tröscher-Mußotter et al. https://creativecommons.org/licenses/by/4.0/ This content is distributed under the terms of the Creative Commons Attribution 4.0 International license.

### Community dispersion and alpha-diversity cause the formation of distinct microbiome clusters.

Changes in nutritional and physiological conditions over a long time period are significant factors to change the microbial composition. But the extent of such dynamic changes may vary at the individual animal level. This was calculated without considering any *a priori* groups and using the multivariate dispersion (MVDISP) approach. Each animal received a dispersion score depending on its respective samples. This allows for the separation of the herd into a dynamic and a static group (Fig. 2b) by using the mean MVDISP of 1.000 as the separator. High MVDISP values indicate high heterogeneity between the samples and a more variable bacterial composition, while low values indicate low heterogeneity. As a result, 26 individuals were assigned to the dynamic group (MVDISP > 1.000) and 28 to the static group (MVDISP ≤ 1.000) over the entire sampling period. In addition, the alpha-diversity calculated as the Shannon diversity index matches for the majority of samples the calculated dispersions, as higher Shannon indexes (≥5.20) are identified for dynamic animals and lower indexes (<5.19) for static animals (Fig. 2c; see Data Set S3 at https://github.com/SebasSaenz/Troscher-Mussotter_Cow-enterotypes_2021/tree/main/Datatsets).

### Clusters of distinct community composition types are identified and linked to dispersion and diversity.

**FIG 3:**
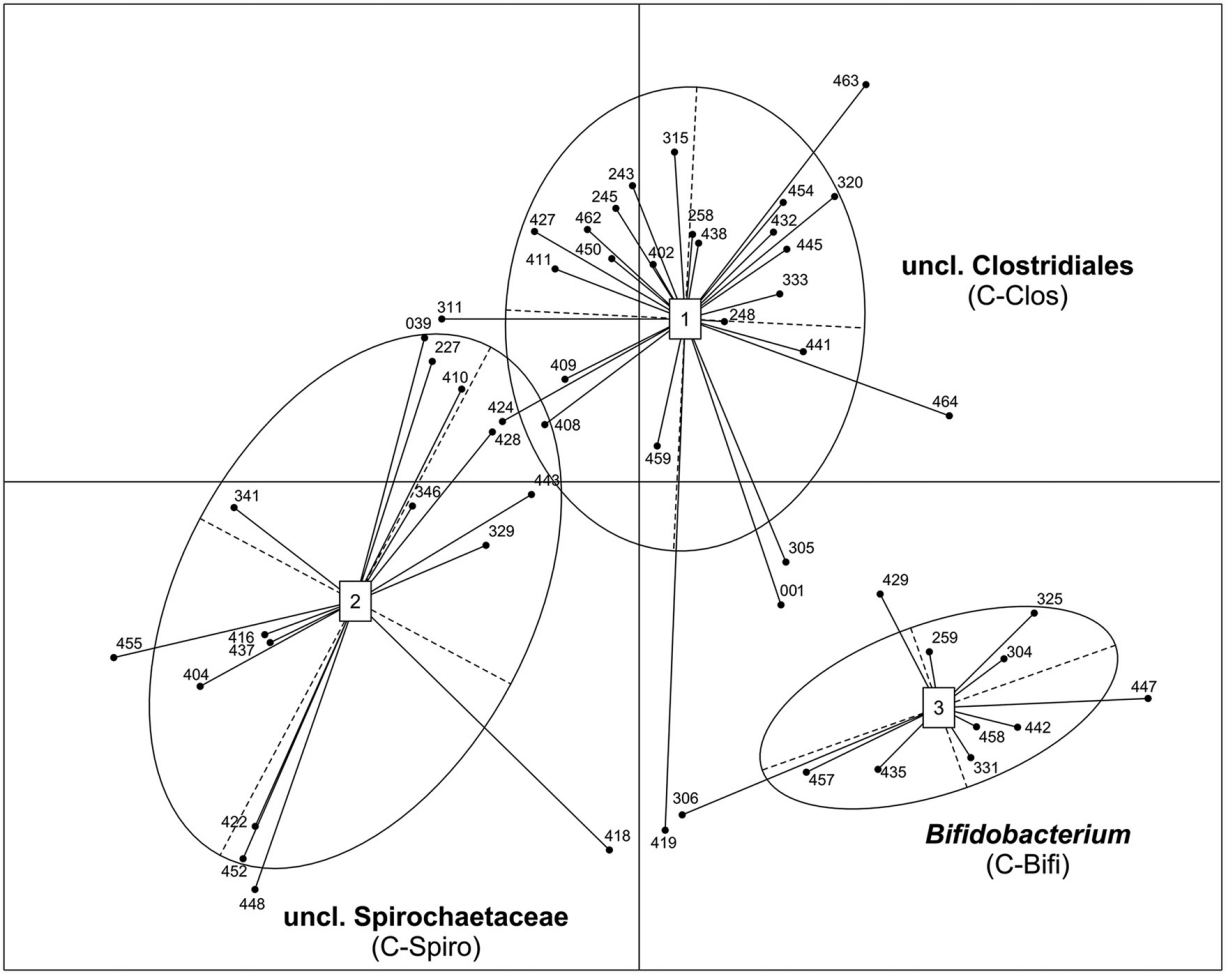
Three clusters of dairy cows within the same herd. Relative abundance genus data were averaged per animal across all 13 time points, resulting in a total of 54 data points included in this R-supported analysis. Labels in the figure indicate the genera with the highest taxon weight of the cluster, as follows: for cluster 1, it was uncl. *Clostridiales* (C-Clos, *n* = 27, CAR:CON = 12:15), for cluster 2, it was uncl. *Spirochaetaceae* (C-Spiro, *n* = 16, CAR:CON = 6:10), and for cluster 3, it was *Bifidobacterium* (C-Bifi, *n* = 11, CAR:CON = 7:4).

Due to the observed differences in dynamics, the hypothesis was that 54 animals could be grouped into distinct community composition clusters for the whole experimental period. The decision to average the data sets per cow was done based on our intention to identify microbiomes which can be linked to animals with good performance and health rather than to describe microbial changes induced by nutrition. Therefore, the enterotype approach of Arumugam et al. (14) was applied, and three distinct microbiome clusters across the herd and experimental period were revealed (Fig. 2d and 3) (PERMANOVA *F* = 9.10, *P = *0.0001). Eleven animals were assigned to the cluster governed by *Bifidobacterium* (C-Bifi) (CAR:CON ratio = 7:4), revealing *Bifidobacterium*, unclassified (uncl.) *Coriobacteriales*, and uncl. *Lachnospiraceae* as the dominant genera (SIMPER average similarity at genus level = 58.4%) (see Data Set S4 at https://github.com/SebasSaenz/Troscher-Mussotter_Cow-enterotypes_2021/tree/main/Datatsets). The cluster governed by the uncl. *Clostridiales* (C-Clos), which comprised 27 animals (CAR:CON = 12:15), was colonized by uncl. *Clostridiales*, uncl. *Ruminococcaceae*, and *Oscillibacter* (SIMPER average similarity at the genus level = 62.4%). Sixteen animals were dominated by uncl. *Spirochaetaceae*, uncl. *Bacteroidetes*, and uncl. *Haloplasmatales* and clustered into the uncl. *Spirochaetaceae* (C-Spiro) group (SIMPER average similarity at genus level = 65.1%; CAR:CON = 6:10). C-Spiro and C-Bifi genus communities were the most disparate across all time points (ANOSIM global *R* = 0.334, *P = *0.0001; SIMPER average dissimilarity at genus level = 44.2%), followed by C-Bifi versus C-Clos (ANOSIM global *R* = 0.264, *P = *0.0001; SIMPER average dissimilarity at genus level = 44.0%) and C-Spiro versus C-Clos (ANOSIM global *R* = 0.079, *P = *0.0001; SIMPER average dissimilarity at genus level = 38.3%). Combining MVDISP and the microbiome cluster results in 91% C-Bifi, 48% C-Clos, and 19% C-Spiro animals, which are sorted into the dynamic group (see Fig. S2 in the supplemental material). Hence, microbiomes of the C-Bifi animals were largely dynamic and those of C-Spiro were largely static, with C-Clos representing an intermediate cluster.

10.1128/mSystems.00856-21.2 FIG S2 Mosaic plot for MVDISP classification on the genus level across the microbiome clusters C-Bifi (*n* = 129), C-Clos (*n* = 307), and C-Spiro (*n* = 174). Dynamic (*n* = 26) and static animals (*n* = 28) were represented equally across animals. The average MVDISP value across all animals was used as a separator between “dynamic” (≥1.000) and “static” (<1.000) animals. Download FIG S2, TIF file, 0.1 MB. Copyright © 2021 Tröscher-Mußotter et al. 2021 Tröscher-Mußotter et al. https://creativecommons.org/licenses/by/4.0/ This content is distributed under the terms of the Creative Commons Attribution 4.0 International license.

**FIG 4:**
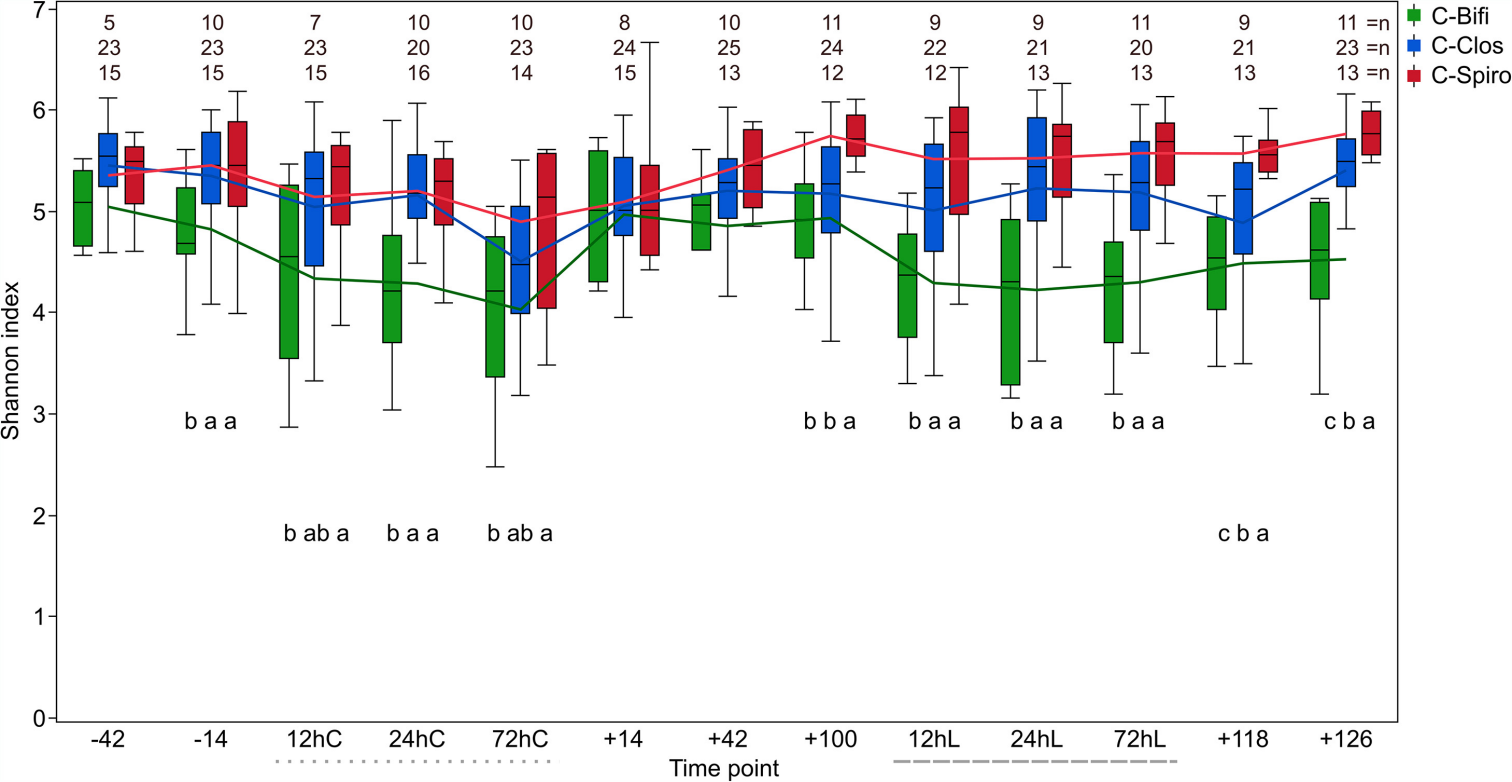
Microbiome clusters with distinct microbial alpha-diversity indices. Shannon diversity indices at the OTU level across 13 time points are given as box plots for each of the three animal clusters. Time points including a “–” or “+” indicate days antepartum or postpartum, respectively, and time points including “hC” or “hL” are samples taken at 12, 24, or 72 h after calving (dotted line) or LPS challenge (dashed line), respectively. Letters below boxes indicate significance by the nonparametric Wilcoxon test (*P ≤ *0.05); levels not labeled with the same letter are significantly different. *n* values are the numbers of cows included per time point and cluster.

Copy number counts of quantitative PCR (qPCR)-derived total bacteria are only significantly different at time point +14 with C-Clos, showing a significantly higher count than the other clusters (Fig. S3a). Diversity across individual time points is significantly higher in C-Spiro, followed by C-Clos, and lowest in C-Bifi (Fig. 4). A significant decrease in diversity during calving and LPS challenge was observed to different extents. Regression slopes indicated a stronger significant decrease in diversity during calving (−42 to 72hC) in C-Bifi (*R*^2^ = 0.2, *P = *0.004) and C-Clos (*R*^2^ = 0.2, *P = *0.0001) than in C-Spiro (*R*^2^ = 0.1, *P = *0.02). The decreasing slope across the LPS challenge (time point +100 to 72 h after LPS challenge [72hL]) was significant only for C-Bifi (*R*^2^ = 0.1, *P = *0.03). ANOSIM analysis reveals significantly stronger effects due to microbiome clusters than to treatment (see Data Set S4 at https://github.com/SebasSaenz/Troscher-Mussotter_Cow-enterotypes_2021/tree/main/Datatsets).

10.1128/mSystems.00856-21.3 FIG S3 qPCR results as box plots with error bars for the three microbiome clusters at different time points for total copy numbers of bacteria (a) and *Bifidobacterium* species (b). Download FIG S3, TIF file, 0.4 MB. Copyright © 2021 Tröscher-Mußotter et al. 2021 Tröscher-Mußotter et al. https://creativecommons.org/licenses/by/4.0/ This content is distributed under the terms of the Creative Commons Attribution 4.0 International license.

### Bacterial networks in the microbiome clusters.

The main discriminative genus between the microbiome clusters is *Bifidobacterium*, which largely contributes to the total dissimilarity between the three animal clusters (SIMPER average, 15.9%) (see Data Set S4 at https://github.com/SebasSaenz/Troscher-Mussotter_Cow-enterotypes_2021/tree/main/Datatsets). It shows the highest relative abundance in C-Bifi (14.6%), followed by C-Clos (8.6%) and C-Spiro (6.9%) (Fig. S4 and S5). Copy numbers of *Bifidobacterium* matched Illumina sequencing results in trends (Fig. S6) but are not significantly different between the clusters across different time points (Fig. S3b). Members of uncl. *Bacteroidales* were highest in C-Bifi (28.4%) in comparison to the other two clusters (C-Clos, 23.1%; C-Spiro, 24.2%) and contributed an average of 13.2% to dissimilarity among them. Uncl. *Lachnospiraceae* members were highest in C-Bifi (14.0%), followed by C-Clos (9.4%) and C-Spiro (7.9%), contributing an average of 9.2% to the dissimilarity. Uncl. *Clostridiales* members were the most abundant in C-Clos animals (15.8%), followed by C-Spiro (12.7%) and C-Bifi (9.9%), contributing an average of 8.2% to cluster dissimilarity. *Turicibacter* members were lowest in C-Bifi (0.6%) and C-Spiro (2.9%) and the highest in C-Clos (4.1%), contributing an average of 4.6% to their dissimilarity. Uncl. *Ruminococcaceae* members contributed an average of 5.4% to the cluster separation, with higher abundance in C-Clos (9.8%), followed by C-Spiro (8.0%) and C-Bifi (5.9%).

10.1128/mSystems.00856-21.4 FIG S4 PCoA plot of relative abundance data for genera. The three clusters were labeled C-Bifi (green), C-Clos (blue), and C-Spiro (red). Spearman’s correlation vectors indicate *Bifidobacterium* and uncl. *Bacteroidales* with an *r* of ≥0.8, *Turicibacter* with an *r* of ≥0.7, and uncl. *Clostridiales* and uncl. *Ruminococcaceae* with an *r* of ≥0.65. Download FIG S4, TIF file, 0.2 MB. Copyright © 2021 Tröscher-Mußotter et al. 2021 Tröscher-Mußotter et al. https://creativecommons.org/licenses/by/4.0/ This content is distributed under the terms of the Creative Commons Attribution 4.0 International license.

10.1128/mSystems.00856-21.5 FIG S5 Heat maps of the main genera involved in separation between the microbiome clusters C-Bifi (*n* = 120), C-Clos (*n* = 292), and C-Spiro (*n* = 179) at all 13 time points and relative abundance (%) in 54 cows. Time points including a “–” or “+” indicate days antepartum or postpartum, respectively, and time points including “hC” or “hL” are samples taken at 12, 24, or 72 h after calving or LPS challenge, respectively. *n* values are the total number of samples averaged among cluster animals per time point. Within-heat-map letters indicate nonparametric Wilcoxon test significance (*P ≤ *0.05). Download FIG S5, TIF file, 0.2 MB. Copyright © 2021 Tröscher-Mußotter et al. 2021 Tröscher-Mußotter et al. https://creativecommons.org/licenses/by/4.0/ This content is distributed under the terms of the Creative Commons Attribution 4.0 International license.

10.1128/mSystems.00856-21.6 FIG S6 *Bifidobacterium* abundances as a heat map for the three microbiome clusters at different time points sequenced using Illumina sequencing (relative abundance, %) (a) and qPCR (total copy numbers of *Bifidobacterium* species) (b). Download FIG S6, TIF file, 0.2 MB. Copyright © 2021 Tröscher-Mußotter et al. 2021 Tröscher-Mußotter et al. https://creativecommons.org/licenses/by/4.0/ This content is distributed under the terms of the Creative Commons Attribution 4.0 International license.

**FIG 5:**
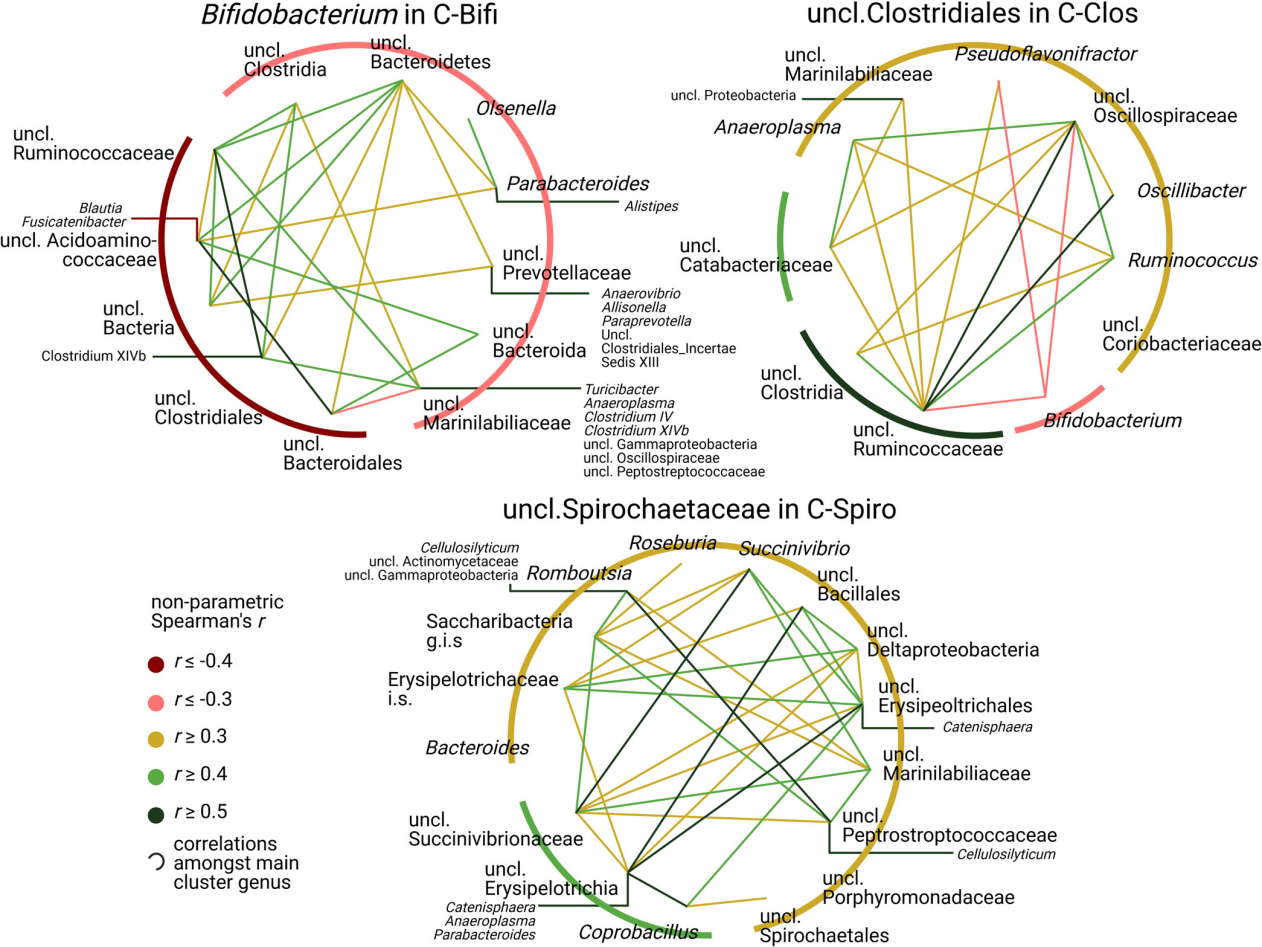
Microbiome clusters are defined by divergent correlations among community members. Circular correlation networks including 591 samples were drawn from nonparametric Spearman’s rank analysis (all significant at a *P *of *≤*0.006) of microbiome clusters for their respective dominating genera: *Bifidobacterium* (C-Bifi), uncl. *Clostridiales* (C-Clos), and uncl. *Spirochaetaceae* (C-Spiro). Open circle shapes with overlying taxon names indicate first-level correlations with the respective dominating genus. Inner-circle lines indicate genera with second-level correlations. From circles, outwardly directed lines indicate third-level correlating genera (solely |*r*| ≥ 0.5), which are not directly connected to the respective dominating genus at a similar strength. For example, *Bifidobacterium* in C-Bifi individuals is at first rank negatively correlating with *Olsenella* (*r  *≤ −0.3), which in turn was positively correlated at the second level with *Parabacteroides* (*r* ≥ 0.4). *Parabacteroides* was at the third level positively correlating with *Alistipes* (*r* ≥ 0.5), for which no direct correlation with *Bifidobacterium* was observed. Correlations were calculated using JMP Pro 15.2.1, and the most important correlations were selected and used to draw the plots.

Cooccurrence network analysis by nonparametric Spearman’s rank correlation (*r*) offers insights into the main interactions between the dominant cluster genera (*Bifidobacterium*, uncl. *Clostridiales*, and uncl. *Spirochaetaceae*) and other community members at three levels of interaction (Fig. 5). In Fig. 5, open circle shapes with overlying genera indicate first-level correlations with the respective dominating genus. The inner circle lines indicate genera with second-level correlations. From circles, outwardly directed lines indicate third-level correlating genera (solely |*r*| ≥ 0.5), which are not directly connected to the respective dominating genus with similar strength. *Bifidobacterium* was negatively associated with first-level genera in the C-Bifi and C-Clos clusters but also with uncl. *Ruminococcaceae* at the second level. Uncl. *Marinilabiliaceae* was a first-level member in all clusters; however, it was negatively associated with *Bifidobacterium* in C-Bifi (*r  *≤ * −*0.3) and positively associated with both of the other clusters (*r* ≥ 0.3). This genus concatenated a wide range of positively correlated genera, such as uncl. *Proteobacteria* in C-Clos (third level), as well as uncl. *Peptostreptococcaceae* and *Succinivibrio* in C-Spiro (second level). Uncl. *Spirochaetaceae* in C-Spiro showed an enhanced number of positive correlations with genera at the selected threshold compared to the other clusters.

### Fermentation products and microbiome clusters.

Short-chain fatty acids (SCFAs) of 610 fecal samples of 54 animals, covering 13 time points, were measured (Fig. S7; see Data Set S5 at https://github.com/SebasSaenz/Troscher-Mussotter_Cow-enterotypes_2021/tree/main/Datatsets). Generally, time had a more significant impact on SCFA (ANOSIM global *R* = 0.207, *P = *0.0001; PERMANOVA *F* = 21.5, *P = *0.0001) than treatment (ANOSIM global *R* = 0.008, *P* = 0.048; PERMANOVA *F* = 5.4, *P = *0.007) or microbiome clusters (ANOSIM global *R* = 0.007, *P = *0.265; PERMANOVA *F* = 0.4, *P = *0.83). During early lactation, C-Spiro showed a rapid and enhanced formation of propionate, butyrate, isobutyrate, valerate, and isovalerate. However, during LPS challenge at time point 12hL, clusters C-Bifi and C-Clos formed higher concentrations of propionate, isobutyrate, valerate, and isovalerate, with a delay for higher acetate concentrations at 72hL.

10.1128/mSystems.00856-21.7 FIG S7 Heat maps of fecal SCFA (mM, *n* = 610) of dairy cows divided into microbiome clusters C-Bifi (*n* = 129), C-Clos (*n* = 307), and C-Spiro (*n* = 174) from 42 days antepartum (−42) to 126 days postpartum (+126). Time points including a “–” or “+” indicate days antepartum or postpartum, respectively, and time points including “hC” or “hL” are samples taken at 12, 24, or 72 h after calving or LPS challenge, respectively. *n* values are the total number of samples averaged per time point. Within-heat-map letters indicate nonparametric Wilcoxon test significance (*P ≤ *0.05). Download FIG S7, TIF file, 0.2 MB. Copyright © 2021 Tröscher-Mußotter et al. 2021 Tröscher-Mußotter et al. https://creativecommons.org/licenses/by/4.0/ This content is distributed under the terms of the Creative Commons Attribution 4.0 International license.

### Fecal metabolites and microbiome clusters.

**FIG 6:**
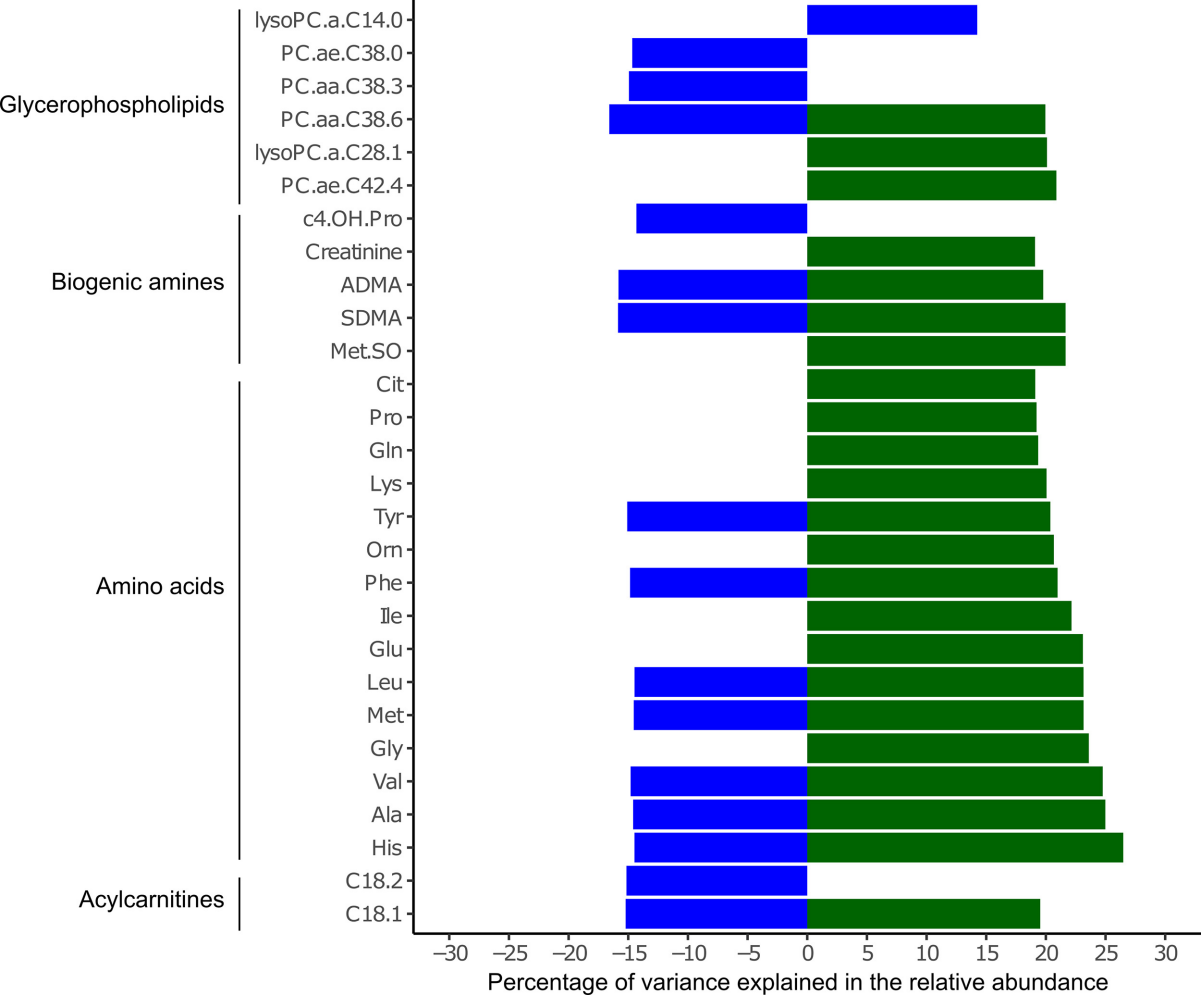
Microbiome clusters are differentially associated with fecal metabolites. Percentages of variance in relative abundance of *Bifidobacterium* (green) and uncl. *Clostridiales* (blue) are given for 275 metabolite samples and 47 animals, with significantly associated metabolites (FDR-corrected *P < *0.05) derived from the Biocrates p180 panel.

A metabolomics approach targeting 188 different metabolites is used to obtain additional information about the host and microbiome function, with respect to time, challenges, and composition (see Data Set S5 at https://github.com/SebasSaenz/Troscher-Mussotter_Cow-enterotypes_2021/tree/main/Datatsets). A subset of seven time points was chosen to cover the two challenge phases in all animals. Time had a strong impact on metabolite composition (ANOSIM global *R* = 0.102, *P = *0.0001; PERMANOVA *F *= 11.10, *P = *0.0001) compared to microbiome clusters (ANOSIM global *R* = −0.002, *P = *0.526; PERMANOVA *F *= 3.65, *P = *0.0001) and treatment (ANOSIM global *R* = 0.016, *P = *0.007; PERMANOVA *F *= 3.15, *P = *0.03). The dominant metabolite groups in the fecal samples were amino acids (>57%) and hexoses (H1) (>31%), followed by biogenic amines (>4%), with alanine (Ala), glutamine (Glu), and glycine (Gly) as major metabolites, which were all highest in C-Bifi. The total fecal metabolite concentration is significantly higher in C-Bifi than in C-Spiro (*P = *0.016), with amino acid concentrations contributing the most to this difference (Fig. S8) (*P = *0.04). *Bifidobacterium* abundance is significantly positively associated with higher levels of histamine (His), Ala, valine (Val), Gly, methionine (Met), leucine (Leu), Glu, isoleucine (Ile), methionine sulfoxide (Met-SO), and symmetric dimethylarginine (SDMA) (Fig. 6). Uncl. *Clostridiales* was negatively correlated with phosphatidylcholine diacyl C38:6 (PC.aa.C38.6), asymmetric dimethylarginine (ADMA), SDMA, C_18:2_, His, Ala, Val, Met, and Leu but positively correlated with lysophosphatidylcholine C14:0 (lysoPC.a.C14.0). The uncl. *Spirochaetaceae* genera did not show significant correlations with the metabolites. Four lysophatidylcholines (lysoPC.a.C14.0, lysoPC.a.C16.0, lysoPC.a.C17.0, and lysoPC.a.C24.0) were significantly (*P < *0.05) higher in dynamic animals than in static animals. Phosphatidylcholines (PC), irrespective of microbiome clusters, showed a clear gap between time points before 12hC (higher PC.aa.C42.2, Spearman’s *r* ≥ 0.8) and after 72hC. Amino acids, irrespective of microbiome clusters, revealed a separation between time points before 12hL and after 72hL (higher His, threonine [Thr], and tryptophan [Trp]; all Spearman’s *r* ≥ 0.7).

10.1128/mSystems.00856-21.8 FIG S8 Heat maps of fecal metabolite groups in total concentration (μmol/liter) by microbiome cluster C-Bifi (*n* = 65 samples), C-Clos (*n* = 148 samples), and C-Spiro (*n* = 80 samples) of a total of 54 cows. Time points including “–” or “+” indicate days antepartum or postpartum, respectively, and time points including “hC” or “hL” are samples taken at 12, 24, or 72 h after calving or LPS challenge, respectively. *n* values are the total number of samples averaged per time point. Within-heat-map letters indicate nonparametric Wilcoxon test significance (*P ≤ *0.05). Download FIG S8, TIF file, 0.1 MB. Copyright © 2021 Tröscher-Mußotter et al. 2021 Tröscher-Mußotter et al. https://creativecommons.org/licenses/by/4.0/ This content is distributed under the terms of the Creative Commons Attribution 4.0 International license.

### Functional prediction of the microbiome clusters.

Rumen-specific functional prediction of the gut microbiome was performed using CowPI precalculated files and PICRUSt. In total, 256 KEGG pathways were predicted among all samples, and 17 amino acid pathways were manually selected as metabolome analyses, indicating differences in amino acid profiles. The analysis reveals significant differences primarily in amino acid-related pathways between the microbiome clusters (Fig. S9). Ala, aspartate (Asp), Glu, cysteine (Cys), Met, and tyrosine (Tyr) metabolism and biosynthesis of amino acid-related enzymes lysine (Lys), Val, Leu, and Ile are increased in C-Bifi. C-Spiro was higher in amino and nucleotide sugar metabolism, and C-Clos was higher in aminobenzoate degradation, d-alanine metabolism, and Val, Leu, and Ile degradation.

10.1128/mSystems.00856-21.9 FIG S9 CowPI predictions including 275 metabolite samples and 177 metabolites of 47 cows by microbiome clusters at various amino acid metabolism traits. FDR *P* values are given if significant differences occurred among all clusters. Download FIG S9, TIF file, 0.4 MB. Copyright © 2021 Tröscher-Mußotter et al. 2021 Tröscher-Mußotter et al. https://creativecommons.org/licenses/by/4.0/ This content is distributed under the terms of the Creative Commons Attribution 4.0 International license.

### Microbiome clusters are linked to animal’s health records and production parameters.

**FIG 7:**
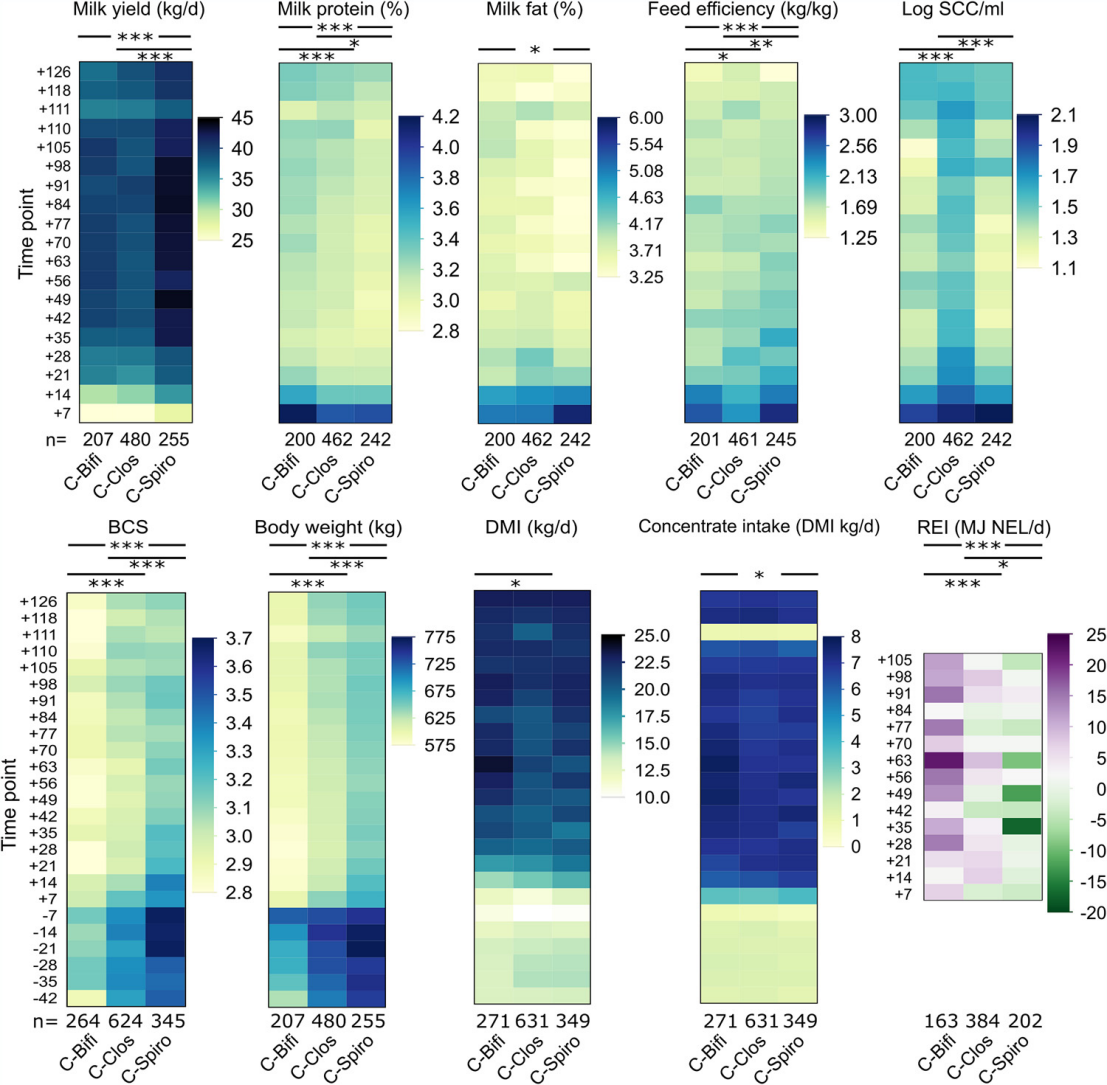
Microbiome clusters show differences in production and physiological parameters across multiple time points. Significant differences among microbiome clusters C-Bifi (11 animals), C-Clos (27 animals), and C-Spiro (16 animals) are indicated by *P* values (***, *P ≤ *0.0001; **, *P ≤ *0.005; *, *P ≤ *0.05) derived from the nonparametric Wilcoxon method. *n* values are the total number of samples averaged per cluster. Time points including a “−” or “+” indicate days antepartum or postpartum, respectively, and time points including “hC” or “hL” are samples taken at 12, 24, or 72 h after calving or LPS challenge, respectively. SSC, somatic cell count; BCS, body condition score; DMI, dry matter intake; REI, residual energy intake.

The long-term experimental trial, including calving and inflammatory challenge, affected animal health to different extents. Results of daily visual examination of dairy cows are shown in Data Set S6 at https://github.com/SebasSaenz/Troscher-Mussotter_Cow-enterotypes_2021/tree/main/Datatsets and were evaluated with respect to the microbiome clusters and the corresponding animals. Seventy-two percent of C-Bifi animals were ill at least once, struggling with one or more illnesses 6.1 days on average (standard error of the mean [SEM], 2.4 days). Seventy-four percent of C-Clos animals were sick 11.4 days on average (SEM, 3.2 days), compared to 81% of all C-Spiro animals, which were sick 9.8 days on average (SEM, 5.3 days). Animals belonging to C-Clos experienced a broader spectrum of health issues, followed by C-Spiro and C-Bifi. Milk yields, body condition scores (BCS), body weight (BW), and residual energy intake (REI) are significantly higher (*P ≤ *0.0001) in C-Spiro animals than in C-Bifi animals (Fig. 7; see Data Set S7 at https://github.com/SebasSaenz/Troscher-Mussotter_Cow-enterotypes_2021/tree/main/Datatsets). In turn, C-Bifi animals had significantly higher concentrate intakes, REI values, and milk protein (*P ≤ *0.0001) and fat (*P ≤ *0.05) concentrations than C-Clos animals. C-Clos animals had significantly higher milk somatic cell counts (SCC) than both the other clusters, significantly lower dry matter intakes than C-Bifi (*P ≤ *0.05), and were mostly intermediate for the other parameters. Rectal temperatures were significantly higher at 3 days antepartum for C-Bifi animals than C-Spiro animals (*P = *0.0293) (see Data Set S6 at https://github.com/SebasSaenz/Troscher-Mussotter_Cow-enterotypes_2021/tree/main/Datatsets).

### Influence of microbiome clusters on blood parameters.

Blood samples were collected at 37 time points, and 23 time points were included in the present study, covering 62 metabolites and cellular parameters (see Data Set S8 at https://github.com/SebasSaenz/Troscher-Mussotter_Cow-enterotypes_2021/tree/main/Datatsets). The effects of time point and treatment on red blood cell count (15), energy metabolism, and electrolytes (16, 17) have recently been published. Among all the parameters, chloride (Cl) is significantly different (*P < *0.0001) between each microbiome cluster, with the highest significance in C-Bifi and the lowest in C-Spiro (Fig. S10). No significant differences in water intake among the microbiome clusters were observed. In addition, ionized calcium (Ca^2+^) and hematocrit (HCT; measured using Celltac) were significantly different (*P ≤ *0.0001) between each cluster, with the lowest significance for C-Bifi and the highest for C-Spiro. Mean platelet volumes (MPV) were significantly different (*P < *0.0001) between all three clusters, with that of C-Clos at the highest and that of C-Bifi at the lowest level. In contrast, C-Bifi had significantly higher carnitine and gamma-butyrobetaine (gBB) concentrations and ferric reducing ability of plasma (FRAP) (*P ≤ *0.02). In addition, this cluster showed a significantly lower mean corpuscular volume (MCV), mean corpuscular hemoglobin (MCH), glutathione peroxidase (GPx), thrombocrit (PCT), partial pressure of carbon dioxide (TpCO_2_, temperature corrected), partial carbon dioxide pressure (pCO_2_), hemoglobin (HGB), total carbon dioxide (tCO_2_), bicarbonate (HCO_3_), and non-esterified fatty acids (NEFA) than the other two clusters. For the C-Spiro cluster, blood glucose, lactate, sodium (Na), hematocrit (cHct, measured using blood gas analyzer), and superoxide dismutase (SOD) were significantly lower, and trimethyllysine (TML), triglycerides (TG), and beta-hydroxybutyrate (BHB) concentrations were significantly higher than that of the other clusters. Lymphocytes (LYP) were significantly lower, and absolute granulocytes (GR), neutrophile granulocyte percentage (GRP), and white blood cells (WBC) were significantly higher in C-Clos animals than in the other two clusters.

10.1128/mSystems.00856-21.10 FIG S10 Blood parameters and platelets according to microbiome clusters C-Bifi (B), C-Clos (C), and C-Spiro (S). *P* values indicate nonparametric and nonpairwise Wilcoxon test significance. For example, mean platelet volumes (MPV) were significantly different between all three microbiome clusters and highest for C-Clos, and TML was significantly higher in C-Spiro than in either other cluster at a *P* value of 0.0001. Ca^2+^, ionized calcium; HCT, hematocrit (Celltac); Cl, chloride; MCV, mean corpuscular volume; MCH, mean corpuscular hemoglobin; GPx, glutathione peroxidase; TpCO_2_, carbon dioxide partial pressure (corrected by temperature); pCO_2_, carbon dioxide partial pressure; HGB, hemoglobin; tCO_2_, total carbon dioxide; HCO_3_, bicarbonate; NEFA, nonesterified fatty acid; gBB, gamma-butyrobetaine; FRAP, ferric reducing ability of plasma; GR, neutrophilic granulocytes, absolute; GRP, neutrophilic granulocytes, proportion; WBC, leukocytes (absolute); LYP, lymphocytes (proportion); SOD, superoxide dismutase; Na, sodium; cHct, hematocrit (blood gas analyzer); TML, trimethyllysine; TG, triglycerides; BHB, beta-hydroxybutyrate. Download FIG S10, TIF file, 0.2 MB. Copyright © 2021 Tröscher-Mußotter et al. 2021 Tröscher-Mußotter et al. https://creativecommons.org/licenses/by/4.0/ This content is distributed under the terms of the Creative Commons Attribution 4.0 International license.

## DISCUSSION

The aim of this long-term study was to elucidate the relationship between the dairy cow as a host, its fecal metabolites and microbiome during two challenging periods, and the modulating ability of l-carnitine. Combining these data with blood metabolite and performance data offered insights into different stages of the dairy cow’s life and revealed unknown relationships between the above-mentioned players. l-Carnitine supplementation did not affect fecal metabolites or bacterial consortia in the present study. However, CAR animals had higher milk fat and serum triacylglyceride concentrations (16), as well as increased blood platelets and plateletcrit (15) during early lactation. In addition, higher insulin levels and lower NEFA concentrations were observed after LPS injection (17).

As a major novel finding, this study detected three “robust clusters,” as previously described in humans and referred to as “enterotypes” (14). Enterotypes are the strongest separator for microbial community structures in murine models (18) yet have never been reported in dairy cows. The respective microbiome clusters were characterized by different community structures, microbial diversity, fecal SCFA, and metabolite patterns. In addition, the animal’s health conditions and performance data were cluster specific. As previously observed, enterotype distributions varied over time instead of underlying a continuum (19). Hence, it was decided to refer to them as “microbiome clusters” in the present study.

Finding the “ideal” (20) or “steady state” of the microbiome is a goal in human and livestock animal studies (21, 22), as it is seen as the “healthy” mode. High stability may be beneficial in keeping up inherent functional relationships between the host and its microbiome (23). However, in the case of strong environmental disturbances, the complete system may be at a higher risk of collapse (23). The implications of flexible microbiomes, defined by quick losses and gains of taxa, are receiving more attention. However, the response potential of the host microbiome to environmental changes has not yet been explored (23). Small sample sizes and the lack of knowledge of physiological fluctuations of microbial communities often do not allow classification into dynamic or static phenotypes. Therefore, the present study offers new insights into the response of static and dynamic dairy cow microbiomes to different challenges. It was observed that animals with a dynamic microbiome had low fecal alpha-diversities and positive REI values, consistent with the results of previous studies on steers, where high fecal alpha-diversities correlated with low residual feed intake (RFI) (24). Animals with a dynamic microbiome, mostly associated with the C-Bifi group, were observed to have higher milk quality (milk LogSCC, protein, and fat), lower body weight, fewer health issues, and lower ketosis risk (low NEFA, BHB, and TG; high glucose and carnitine). Therefore, it is suggested that animals with a dynamic microbiome might have a lower risk of ketosis and fatty liver disease. Further research on the flexibility of microbiomes should be conducted to evaluate static or dynamic microbial communities and their implications for the host.

At the start of the lactation period, fiber is considerably replaced by readily fermentable carbohydrates to meet the energy demand of the dairy cow. This increases ruminal SCFA concentrations (6) for energy coverage and subsequent milk production, as well as microbial biomass in feces (25). Most microbial proteins are absorbed in the small intestine but also enter the large intestine, where they can be implicated in microbial cross talk of residential microbes (26). *Bifidobacterium* and other fiber-degrading bacteria can thrive in the hindgut with the remaining microbial proteins and metabolites, complex fibers, and host glycoproteins as nutrient sources. The protein sources were further digested or used for *de novo* synthesis of free amino acids. In the present study, concentrations of amino acids in the feces during transition increased with increasing SCFA concentrations. Therefore, high-grain feeding simplified the cascade of digesta degradation, accelerated its passage rate, and presented postruminal sections with a highly degradable substrate (9, 25). The physiological purpose of gastrointestinal compartments is hereby suspended, and formerly specialized consortia cannot apply their functions (1). This opens the niche to a more diverse set of invading bacteria, such as facultative anaerobic *Proteobacteria* (27). *Proteobacteria* are involved in inflammaging processes and leaky gut syndrome in mice (28). In the present study, *Proteobacteria* members were 4.5-fold more abundant in static C-Spiro animals than in dynamic, low-alpha-diversity C-Bifi animals. Interestingly, the latter cluster was found to struggle with fewer health issues than the C-Clos cluster, which, however, also included the most individuals. As a result, this may lead to an overinterpretation of the clusters’ disease status. Cluster C-Clos covered a much broader range of more cost-intensive health impairments, with the highest milk SCC as well as blood MPV, WBC, and granulocytes, which are involved in the immune response during infections and may indicate a disruption of the gut intestinal barrier (12). C-Clos animals had higher *Turicibacter* abundances, which were positively associated with high-grain feeding and cecal mucosa damage via interleukin 6 (IL-6) and IL-12 mRNA expression in goats (29). This may indicate that enterotypes may be able to group animals according to their inflammatory status (18). Fecal *Turicibacter* abundances were negatively associated with functional traits such as amino acid metabolism, biosynthesis of secondary metabolites, enzyme families, and lipid metabolism in a previous dairy cow study (30). In the present study, high *Turicibacter* abundance was associated with low concentrations of free fecal amino acids and a lowered abundance of predicted amino acid metabolism, which corroborates previous findings. The respective animal productions were characterized by lower milk protein and milk fat levels. A pronounced increase in *Turicibacter* was observed during the challenging phases for C-Clos and C-Spiro, but *Turicibacter* was almost absent in C-Bifi animals. The findings of Liu et al. (29) combined with the present results may indicate that C-Clos and C-Spiro animals might have struggled with intestinal damage. Whether the increase in *Turicibacter* is the cause or the consequence of possible inflammation needs to be evaluated.

*Clostridiales* are involved in amino acid fermentation in the large intestine and use ethanol and lactate as substrates to produce CO_2_, hydrogen, and SCFA (31), and intestinal CO_2,_ derived from digestive fermentation is absorbed from the small intestine (26, 32). Increased CO_2_ levels in the intestine (hypercapnia) promote hypoxia-inducible factor degradation, which plays a major role in intestinal tight junction integrity and mucus stabilization (33, 34). CO_2_ is also produced during hepatic β-oxidation and stimulates satiety (35). Additionally, high blood pCO_2_ concentrations have been associated with subacute ruminal acidosis (SARA) (36). Here, animals with microbiome clusters having a high abundance of uncl. *Clostridiales* (C-Clos > C-Spiro) and higher blood pCO_2_, and the respective OTUs were mostly linked to the degradation of fecal amino acids rather than biosynthesis. This is usually accompanied by an increased production of fecal branched-chain fatty acids (isobutyrate and isovalerate) (26), which was not observed. In addition, saturation signaling via high blood pCO_2_ levels in animals with high abundance of uncl. *Clostridiales* (C-Clos) are mirrored by significantly lower dry matter intake (DMI) and concentration intake than animals with low abundance of uncl. *Clostridiales* (C-Bifi), thereby supporting the findings of previous studies (35). Together, these results indicate that the microbiome clusters coped differently metabolically and may mirror different health conditions. In addition, high *Turicibacter*, uncl. *Clostridiales*, and uncl. *Ruminococcaeae* abundances may indicate a stiffened and inflexible fecal microbiome with static dispersions and high alpha-diversity. This was detected in the present study in heavy animals, with impaired energy metabolism (e.g., low blood glucose and high TG, NEFA, and BHB), lowered milk quality (LogSCC, fat, protein), and high blood TML concentrations, the precursor of carnitine (37). C-Bifi was observed to have significantly less TML but the highest carnitine and gBB concentrations and vice versa for C-Spiro, therefore suggesting that C-Spiro covered carnitine demands via TML upregulation. Both TML and gBB are known to be involved in cardiovascular diseases (26, 38). The significantly higher concentration of blood carnitine and gBB in C-Bifi animals might be due to an unbalanced CAR:CON animal ratio (7:4), as significantly higher concentrations were observed previously in the same CAR animals (16).

The fecal microbiome of C-Bifi animals showed an increase in *Bifidobacterium* at 3 days postpartum. Other studies on the development of the calf microbiome showed concurrent enrichment with *Bifidobacterium* during the first days after birth (39, 40). The trigger for this common development is unknown. Increased involvement in amino acid traits and high milk protein concentrations may indicate that C-Bifi animals possibly provide their calves with higher amounts of prebiotic glycoproteins and possibly even intact probiotic bifidobacteria via the entero-mammalian pathway (41, 42). Milk glycoproteins promote *Bifidobacterium* in the infant’s gut, which increases fecal acetate production in human infants (43). An appropriate acetate increase was confirmed in the present study and during high *Bifidobacterium* abundance in C-Bifi animals. Cows with a microbiome low in *Bifidobacterium*, as well as the conventional premature separation of the calf from the cow, may pose poor initial conditions for the calf and, as data suggest, for the dam at the onset of lactation. To confirm this connection between bifidobacterial strains and *Bifidobacterium*-promoting and/or derived metabolites, a study up to a minimum of 7 days postpartum should be conducted to examine both calves and dams. Bifidobacteria are the first settlers in the calf’s intestine and produce bacteriocins (44), which may protect against an explosive or adverse establishment of pathogenic bacteria, which bear the risk of diarrhea, the most common cause of early deaths in calf husbandry (45, 46). Bifidobacteria may instead allow a controlled settlement of this bare and sensitive niche in newborns. The same is true for the dam, as bifidobacteria may have protective effects against *Enterobacteriaceae*, obesity during gestation, and ketosis, as observed in women (47). Largely negative associations of *Bifidobacterium* with other bacteria were found in the C-Bifi and C-Clos animals. This may indicate their ability to modulate the microbial community composition in later stages of the cow’s life, possibly via bacteriocin expression. In the present study, animals with lower body weights had higher abundances of *Bifidobacterium*, which has recently been found in human enterotype studies (48). Furthermore, C-Bifi had negative third-level correlations with uncl. *Gammaproteobacteria* compared to the other two clusters. C-Bifi individuals increased BCS and BW just before calving, to almost the same weight at day −7 as both other clusters. This indicates the growth of the fetus in C-Bifi animals, rather than an increase in body mass, which was different in C-Spiro animals. The latter struggled with higher blood TG and NEFA levels at low blood glucose levels compared to C-Bifi animals, which suggests a higher fat mobilization, possibly due to a negative energy balance (5). NEFA contribute to milk fat and energy synthesis via β-oxidation in the liver (5). As milk fat was significantly lower in C-Spiro animals than in C-Bifi animals, NEFA might have largely gone into liver β-oxidation rather than milk fat production. This might have resulted in a higher risk of developing milk fat depression, ketosis, and fatty liver disease in highly productive and “efficient” animals, according to negative REI values (5, 35).

A negative REI has been a breeding target for decades, aiming for highly energy-efficient animals defined by low feed intakes with high milk yields and therefore high profitability (24, 49), yet at high body mass mobilization. Negative REI values and other breeding targets, such as high milk yields with low SCC, were found within the static C-Spiro cluster animals, and herein assorted animals would be labeled as “efficient.” C-Bifi animals, in turn, would be labeled as “inefficient,” due to largely positive REI values (50, 51). However, C-Bifi individuals recovered faster from LPS injection, indicated by an earlier restart of SCFA production, quicker fever recovery (not significant), higher blood glucose levels, and higher milk fat and protein levels, with lower BCS, BW, and average days of illness. Hence, the dilemma of modern dairy cows can be addressed by these findings and should be further elucidated by large cohort studies. Data suggest that grouping dairy cows as “inefficient” and “efficient” according to the REI value needs to be rethought, as “inefficient” animals seem to be those with better fitness. Therefore, breeding positive-REI dairy cows may have unbeneficial outcomes for the cow’s fitness in the long run.

Previous studies have observed enriched enzymes for protein digestion and amino acid biosynthesis in the rumen samples of inefficient animals (51). A similar trend was observed in the C-Bifi animals. However, higher fecal excretion of amino acids points toward the higher environmental pollution potential of C-Bifi animals.

Increased *Bifidobacteriaceae* and a largely negative correlation with other families (e.g., *Ruminococcaceae*) have been previously observed in fecal samples of high-RFI steers, which together with no significant difference in fecal SCFA concentrations between the groups is in accordance with the present data (24). The above-mentioned attributes indicate that higher abundances of *Bifidobacterium* in the large intestine of ruminants might keep the total microbiome more flexible and, hence, with quicker reactions to challenging environmental changes than animals with a more static microbiome.

How is it possible that highly synchronized animals (e.g., parents, diet, reproductive cycle, environment) develop different community structures that have beneficial or harmful health and physiological outcomes? In addition to the supplementation, which did not show strong effects, the trial animals varied by their trial animal history of participation in multiple short- and long-term studies, such as studies of milk replacers, antibiotics, or fungal infestation of feed on the cow’s physiology. These studies were performed at all life stages of a dairy cow: as a calf or a mature cow and during gestation, lactation, and rearing. Bifidobacteria and possibly other bacteria are highly sensitive to antibiotics, and long-lasting impacts have been documented, mainly in humans (52, 53). It is suggested that the identified microbiome clusters may reflect long-term and cumulative effects on manipulated intestinal conditions due to multiple previous trials. As the latest studies discuss host genetic influences on intestinal microbial compositions, it may also be possible that clusters genetically mirror more similar groups. For example, host genetic effects on rumen bacteria have been observed for *Bifidobacterium* and *Proteobacteria* (54).

The enterotype approach has been discussed critically (19). In the present study, the calculation of the microbiome clusters resulted in a loss of taxonomic information, as an average of all genera per cow was used to obtain a robust cluster formation. Accepting this, the study proves that the power of this analysis lies in its integrative association with microbiome, metabolite, health, and milk production data and thus has the strength to concatenate blind ends of existing knowledge in dairy cow science.

This long-term study uncovered three distinct microbiome clusters linked to different characteristics of the animal’s physical conditions and body parameters. Each microbiome cluster coped differently with a challenging calving period and an LPS-induced inflammatory stimulus. They differed significantly in their bacterial dynamics, composition and diversity indexes, health status, body mass, milk, and blood parameters. The same diet and housing resulted in different community structure outcomes, showing that it is not only the feed itself that matters but also what the cow’s individual microbiome makes out of it. It would be of great interest if cows from regular farms also showed microbiome clusters and if breeding cows with fewer health issues and positive REI values would sooner or later result in herds and animals with higher *Bifidobacterium* abundances.

The study demonstrated that it might not be the stable microbiome that animal husbandry should aim for but, rather, dairy cows with a more dynamic microbiome that might be more robust by responding quicker to environmental changes. In the future, the importance of *Bifidobacterium* in lactating dairy cows should be as intensively studied as it is in calves, as this study proved the positive effects of a higher fecal abundance of *Bifidobacterium*. Preserving this bifidobacterial community might be a long-term goal, which may yield rich benefits for animal husbandry.

## MATERIALS AND METHODS

### Animal experiment and sampling.

This study is part of the cooperative project “Mitochondrial functionality in dairy cows” (MitoCow) funded by the German Research Foundation (DFG), including 54 multiparous Holstein Frisian dairy cows ranging between 3 and 7 years of age and grouped into a control (CON; *n* = 30) and a carnitine-supplemented (CAR; *n* = 24) herd. Detailed dietary and nutritional composition and study approaches for the calving period are described by Meyer et al. (16) and for the LPS challenge by Meyer et al. (17). In short, 80% roughage and 20% of concentrate were fed until calving (day 0) and contained the supplements in the concentrate feed. Until day 14 postpartum (pp, time point +14), concentrate amounts were gradually increased up to a ratio of 50:50. This regimen was continued until the end of the trial. Roughage comprised 70% maize silage and 30% grass silage; water was offered *ad libitum*. Samples were taken regularly at 7 a.m. after milking at seven time points, between 42 days antepartum (ap, −42) and 126 days pp (Fig. 1) as well as at 12, 24, and 72 h after the calving (hC) and the LPS challenge (hL). This resulted in 13 sampling time points per cow. Calving functioned as an individual, and the LPS challenge functioned at 111 days postpartum (pp, +111) as a standardized stimulus. At this time point, cows are suggested to be out of negative energy balance, which could interfere with the LPS challenge. Before the LPS injection, the animals were examined by veterinarians in order to confirm their state of health. Each cow received 0.5 μg LPS/kg of body weight, which was applied via the jugular vein to provoke an inflammatory challenge. The cows were headlocked at the feeding table during regular sampling and greatly sampled unlocked during the challenges, to reduce stress. Defecation was awaited, and the feces were collected manually before falling to the ground, using long, disposable gloves and aluminum dishes for temporal storage. The fecal heap was then sampled at three different spots for randomization reasons using a sterile metal spoon and avoiding the top layer due to excessive oxygen exposure. A total of 626 fecal samples were stored at −80°C, and not all samples were included in all analysis. Blood was collected from the external jugular vein by needle puncture or by indwelling catheters for the frequent sampling during the challenges as described by Meyer et al. (17).

### Bacterial DNA extraction and amplification for Illumina sequencing.

Microbial DNA of 616 fecal samples was extracted using the FastDNA spin kit for soil (MP Biomedicals, Solon, OH, USA) in accordance with the manufacturer’s instructions with minimal changes (55). DNA quantity and quality were measured using NanoDrop (Thermo Fisher Scientific, Waltham, MA, USA), and subsequently, the DNA extracts were stored at −20°C. The V1-2 region of the 16S rRNA gene was targeted to construct an amplicon Illumina sequencing library amplified using a two-step PCR approach, similar to that described by Kaewtapee et al. (56). During the first and second PCR, barcodes and indexes were attached to the amplicons using TaKaRa PrimerStar HS DNA polymerase (TaKaRa Bio USA, Inc.) (56). PCR amplicons were verified by agarose gel electrophoresis and normalized using the Sequalprep normalization kit (Thermo Fisher Scientific, Waltham, MA, USA), in accordance with the manufacturer’s instructions. Samples were pooled and purified with a MinElute PCR purification kit (Qiagen). The final DNA concentration was measured using a Qubit 2.0 fluorometer (Invitrogen) and a QuantiFluor double-stranded DNA (dsDNA) system (Promega). Samples were sequenced by 250-bp paired-end sequencing on an Illumina MiSeq.

### Sequencing data analysis and taxonomic assignation.

QIIME2 v.2019 (http://qiime2.org) was used to analyze the obtained raw sequences (57). The default parameters of the pipeline were used to perform quality filtering, trimming, and demultiplexing, resulting in a maximum sequence length of 360 bp. The subsequent data set was dereplicated and denoised, chimeras were removed, and data were merged through DADA2 (58). A prefitted sklearn-based classifier (59) was used for taxonomy assignation, equipping the SILVA database (release 132) (https://www.arb-silva.de/) (60). Filtration of 11,187,005 reads and 19,409 operational taxonomic units (OTU) (97% identity) was performed by cutting those appearing only once across 616 fecal samples (countif = 1), and that summed up in total 2 to 10 counts with a maximum of ≤1,000 reads across all samples (maximum = 2 to 10). Those OTUs appearing in only 11 to 20 samples with ≤100 reads were deleted as well. Data filtering resulted in 9,437,285 total reads, grouped into 3,921 OTUs with an average of 15,941 ± 367 reads per sample. The Ribosomal Database Project (RDP) seqmatch tool was used to identify the closest representative of each OTU (61, 62). Subsequently, the taxonomy levels were assigned by following the threshold cutoff values of Yarza et al. (63). The blastn tool of the National Center for Biotechnology Information (NCBI) was used to specify the uncl. *Firmicutes* and uncl. *Spirochaetaceae* genera for the microbiome cluster analysis.

### Microbiome cluster analysis.

Sequence data of the samples were clustered based on the mean relative genus abundance of 591 samples representing 54 animals (CON, 30; CAR, 24). The mean was calculated based on all samples per individual (≤13 samples) to detect global information of the genera contributing to the respective microbiome clusters during a defined production lifespan in dairy cows. This resulted in one set of abundance data across all genera per cow. Microbiome clusters among the animals were identified as formerly described by Arumugam et al. (14) (https://enterotype.embl.de), including the unclassified taxa. Briefly, a Jensen-Shannon divergence matrix was calculated based on the genus-relative abundance using R v.3.6.1 and the “tidyverse” package (64). Then, the partitioning around medoids clustering algorithm was done with “cluster” (65), and the optimal number of clusters, resulting in three clusters, was assessed using the Calinski-Harabasz index and the Elbow method using “clusterSim” (66) and “factoextra” (67). Finally, “ade4” was used to performed a principal-component analysis (PCoA) of the data and visually explore the clusters (68).

### qPCR.

Nine time points (−42, 12hC, 24hC, 72hC, +14, +100, 12hL, 72hL, and +126) and 11 animals per cluster were randomly selected for quantitative PCR (qPCR) analysis, using the above-mentioned DNA extracts, with a total of 287 samples. By following the principles of Lengowski et al. (69), a pooled DNA sample was used as a “sample-derived DNA standard,” confirming the DNA load using Qubit and Nanodrop. Primer pair products were tested on this pooled DNA standard by using a conventional PCR. According to the method described by Lee et al. (70), a 10-fold serial dilution series of each PCR product with six dilutions was used for generating standard curves. For qPCR, two replicates per sample, two negatives, and three replicates of the standard were run on every plate using a CFX real-time PCR instrument (Bio-Rad). Quantification of bacterial copy numbers was done using primers 338F 5′-ACTCCTACGGGAGGCAG and 805R 5′-GACTACCAGGGTATCTAATCC with a product length of 468 bp. The PCR mix contained 160 nM each primer, 2.3 mM MgCl_2_, 3.2% bovine serum albumin (BSA) (1 mg/1 ml), 1× GoTaq qPCR polymerase mix (Promega), and 1 μl of template undiluted DNA. The following conditions were applied to the samples: initial denaturation at 95°C for 2 min, 40 cycles of denaturation at 95°C (15 s), annealing at 50°C (20 s). followed by 60°C for 15 s (two-step qPCR), and a final elongation at 72°C for 1 min. Thereafter, melting curves were measured with slow heating from 65°C for 5 s to 95°C in 0.5-degree steps. Copy numbers of *Bifidobacterium* were determined using a PCR mixture of 200 nM each primer (Bifido_5 GATTCTGGCTCAGGATGAACGC, Bifido_3 CTGATAGGACGCGACCCCAT) (71), 1× GoTaq qPCR polymerase mix (Promega), and 1 μl of 1:10-diluted DNA template, resulting in a product length of 236 bp. Cycle conditions were equivalent to those for total bacteria; however, after denaturation, an annealing step at 60°C for 1 min was used (one-step qPCR). Total copy numbers per sample were calculated using the standard curves.

### Functional prediction.

Amplicon data of seven time points (−42, 12hC, 72hC, +100, 12hL, 72hL, +126) chosen in accordance with the fecal metabolite analyses (275 samples and 47 animals) were used to perform a functional prediction of the fecal microbiome using CowPI and PICRUSt in Galaxy as described by Wilkinson et al. (72).

### SCFA measurement.

From 610 thawed samples, three aliquots per sample were taken, each weighing 4 g. Samples were homogenized, acidified using sulfuric acid (H_2_SO_4_), and supplemented with 80 mM 2-methylvaleric acid in 50% formic acid as an internal standard. The samples were frozen in an Erlenmeyer flask and incubated using a −30°C ethanol bath under continuous movement. The undissociated fatty acids were distilled with liquid nitrogen under vacuum, and 1 ml of distillate sample was used for the determination of acetic (C_2_), propionic (C_3_), butyric (C_4_), isobutyric (C_4_I), valeric (C_5_), and isovaleric (C_5_I) acid. For the analysis of short-chain fatty acids (SCFA) in the fecal samples, a gas chromatograph (GC) (Hewlett-Packard 6890; Agilent) connected to a fused silica capillary column (HP-FFAP; 25 m by 0.32 mm with a film thickness of 0.5 μm; HP 7683; Agilent) and a flame ionization detector (GC-FID) as described by Wischer et al. (73) was used.

### Metabolomic analysis.

Targeted measurements of metabolites were performed using 293 fecal samples from a subset of 7 time points (−42, 12hC, 72hC, +100, 12hL, 72hL, +126; samples per time point for 39 to 45 animals). Metabolite extraction was done as suggested by Biocrates Life Science AG using a buffer with a high extraction efficiency for amino acids, biogenic amines, acylcarnitines, and hexoses. The buffer comprised 80 ml ethanol (Supelco, LiChrosolv) and 320 ml phosphate buffer (20 mM; Sigma P5244; 0.1 M, pH 7.5 at 25°C) (vol/vol). Two hundred milligrams of thawed sample was mixed on ice with 600 μl of buffer B on a shaker at 200 rpm for 30 min, followed by a centrifugation step for 15 s at 19,000 × *g*. Thereafter, samples were tip sonicated on ice for 5 min at 100% amplitude and at 0.5 duty cycle (Ultrasonic UP50H processor with MS1 sonotrode; Hielscher, Germany). Cell debris, feed, and other particles were precipitated by centrifugation at 800 × *g* for 10 min and at 2°C. The supernatant was centrifuged at 19,000 × *g* for 10 min and 2°C. The clean supernatant was stored at −80°C until measurements within the days after processing were done. Fecal samples were further treated in accordance with the manufacturer’s manual for blood plasma samples. Target metabolomics measurements were done using an AbsoluteIDQ p180Kit (Biocrates Life Science AG, Innsbruck, Austria) according to the manufacturer’s instructions. Quantified metabolites (188) included amino acids (21), biogenic amines (21), hexoses (1), acylcarnitines (40), glycerophospholipids (90), and sphingomyelins (15). The first two groups of metabolites were measured using liquid chromatography-tandem mass spectrometry (LC-MS/MS). All other metabolites were analyzed using a flow injection analysis measurement (FIA)-MS/MS equipping a Sciex 4000 QTRAP (Sciex, Darmstadt, Germany) or Xevo TQ-S Micro (Waters, Vienna, Austria) machine combined with electrospray ionization (ESI). The metabolite measurement was described in detail before (74) and was used with the following adjustments: quantification of the biogenic amines was improved by adding the calibration standard 0.25 to the calibration standard curve. The incubation time with phenyl isothiocyanate was extended by 5 min for improved derivatization of the samples. A nitrogen pressure unit was used to elute the extraction solvent. Then, 50 μl was removed from the filtrate, transferred to a fresh multiwell plate, and diluted with 450 μl of 40% HPLC-grade methanol for LC-MS analysis. For FIA-MS/MS analysis, 10 μl from the filtrate and 490 μl of the mobile-phase solvent were added to a new multiwell plate.

### Blood, health, and milk production parameters.

Heparinized blood samples were analyzed immediately after sampling using a GEM Premier 400 blood gas analyzer (Werfen, Kirchheim, Germany) as previously described by Meyer et al. (16). Total blood cell counts were determined in EDTA blood samples using an automated hematology analyzer (Celltac-α MEK 6450; Nihon Kohden Corporation, Japan). Blood metabolites (nonesterified fatty acids, triglycerides, glucose, beta-hydroxybutyrate) were determined in serum samples by using an automatic clinical chemistry analyzer (Eurolyser CCA 180; Eurolyser Diagnostica GmbH, Salzburg, Austria) (15). Residual energy intake (REI), milk parameters, body weights, and daily visual health examinations by the same veterinarian were recorded and recently published (16, 17). The average number of sick days per sick cow was calculated, ignoring the quantity of multiple health issues at 1 day per individual.

### Statistical analysis of sequencing data.

The total number of reads per sample was standardized by the total. The Bray-Curtis similarity coefficient (75) was used to calculate and visualize similarity matrixes and intersample similarity plots (principal-coordinate analysis [PCoA] plots) using PRIMER-E 6 (Plymouth Marine Laboratory, UK) (76). The alpha-diversity and animal’s microbial flexibility over time were evaluated using the Shannon diversity index and multivariate dispersion indices (MVDISP). The average MVDISP across all animals functioned as a separator between “dynamic” (MVDISP > 1.000) and “static” (MVDISP ≤ 1.000) individuals (see Data Set 3 at https://github.com/SebasSaenz/Troscher-Mussotter_Cow-enterotypes_2021/tree/main/Datatsets). Global *R* and *P* values were generated using one-way analysis of similarity (ANOSIM) and permutational multivariate analysis of variance (PERMANOVA). PERMANOVA (permutations = 9,999) was used to test for significance of time point, age, MVDISP, microbiome cluster, and supplementation. The similarity percentages (SIMPER) tool was applied to find the main contributors of differences between groups. Data distribution was analyzed using the Shapiro test in JMP Pro 15.2.1 software (SAS Institute, NC, USA). A mixed model analysis was used to evaluated the differences between the predicted functional pathways (CowPI), and animals were included as a random effect nested by the microbiome clusters (275 samples representing 47 animals). Briefly, R (v.4.0.2) and tidyverse (64) were used to transform and arrange the data. Additionally, the linear mixed-effects model was calculated with ime4 (77), and the differences between each cluster per amino acid were found using ImerTest (78). Copy numbers of *Bifidobacterium* and total bacteria were obtained by qPCR. The Shapiro test in JMP Pro 15.2.1 software was used to test the normality of distribution of bacteria and metabolites, and the Wilcoxon/Kruskal-Wallis test was used to test for significance. The same software and herein the Bivariate Fit tool were used to draw regression slopes for the calving period (−42 to 72hC) and the LPS challenge (+100 to 72hL), in order to evaluate how strongly each microbiome cluster decreased in alpha-diversity.

### Statistics of metabolomics data.

The Biocrates metabolite data were normalized by using the target value of the mean of quality control 2. JMP Pro 15.2.1 software was used to create graphs and to confirm the nonnormal distribution of metabolites, including SCFA using the Shapiro test. As JMP Pro does not require deletion of values below the detection level, 293 samples and 188 Biocrates-derived metabolites were included. The Wilcoxon/Kruskal-Wallis test was used for evaluation of significance. PERMANOVA and ANOSIM analyses of the metabolomics data set were done using PRIMER-E 6. Working with the Biocrates metabolites in PRIMER-E 6 required removal of samples which included values below the detection level, resulting in 177 fecal metabolites and 275 samples from 47 animals (CAR:CON = 22:25).

### Correlation analyses.

Circular correlation networks on genera across microbiome clusters included 591 samples and were drawn from nonparametric Spearman’s *r* multivariate methods, using JMP Pro 15.2.1 software. Only significant (*P ≤ *0.006) and high correlations (|*r*| ≥ 0.2) between genera were considered. Third-level genera were restricted to an |*r*| of ≥0.5.

The linear mixed-model correlations on genera and fecal metabolites included 275 samples from 47 animals and were corrected by age, l-carnitine supplementation, and time point. Animals were considered a random effect, and only significant correlating metabolites were included in the figure (false discovery rate [FDR]-corrected *P < *0.05). The model was calculated with ime4 (77).

### Data and software availability.

Finally, the sequences were submitted to the European Nucleotide Archive under accession number PRJEB44871. The raw metabolomics data sets are available from the corresponding author on reasonable request. Analyzed data are provided as Data Sets S1 to S8 at https://github.com/SebasSaenz/Troscher-Mussotter_Cow-enterotypes_2021/tree/main/Datatsets. Detailed information on data analyses and codes used are provided at https://github.com/SebasSaenz/Troscher-Mussotter_Cow-enterotypes_2021.

## References

[1] Khafipour E, Li S, Tun HM, Derakhshani H, Moossavi S, Plaizier JC. 2016. Effects of grain feeding on microbiota in the digestive tract of cattle. Anim Front 6:13–19. doi:10.2527/af.2016-0018.

[2] Weiss WP. 2017. A 100-year review: from ascorbic acid to zinc—mineral and vitamin nutrition of dairy cows. J Dairy Sci 100:10045–10060. doi:10.3168/jds.2017-12935. 29153154

[3] De Vries A, Marcondes M. 2020. Overview of factors affecting productive lifespan of dairy cows. Animal 14:s155–s164. doi:10.1017/S1751731119003264. 32024570

[4] Bach A. 2011. Associations between several aspects of heifer development and dairy cow survivability to second lactation. J Dairy Sci 94:1052–1057. doi:10.3168/jds.2010-3633. 21257075

[5] van Knegsel AT, Van den Brand H, Dijkstra J, Tamminga S, Kemp B. 2005. Effect of dietary energy source on energy balance, production, metabolic disorders and reproduction in lactating dairy cattle. Reprod Nutr Dev 45:665–688. doi:10.1051/rnd:2005059. 16285910

[6] Plaizier JC, Danesh Mesgaran M, Derakhshani H, Golder H, Khafipour E, Kleen JL, Lean I, Loor J, Penner G, Zebeli Q. 2018. Review: enhancing gastrointestinal health in dairy cows. Animal 12:s399–s418. doi:10.1017/S1751731118001921. 30139397

[7] Schroeder BO, Birchenough GM, Ståhlman M, Arike L, Johansson ME, Hansson GC, Bäckhed F. 2018. Bifidobacteria or fiber protects against diet-induced microbiota-mediated colonic mucus deterioration. Cell Host Microbe 23:27–40.e27. doi:10.1016/j.chom.2017.11.004. 29276171 PMC5764785

[8] Plaizier J, Krause D, Gozho G, McBride B. 2008. Subacute ruminal acidosis in dairy cows: the physiological causes, incidence and consequences. Vet J 176:21–31. doi:10.1016/j.tvjl.2007.12.016. 18329918

[9] Sanz-Fernandez M, Daniel J-B, Seymour DJ, Kvidera SK, Bester Z, Doelman J, Martín-Tereso J. 2020. Targeting the hindgut to improve health and performance in cattle. Animals 10:1817. doi:10.3390/ani10101817. PMC7600859 33036177

[10] Boyle EC, Finlay BB. 2005. Leaky guts and lipid rafts. Trends Microbiol 13:560–563. doi:10.1016/j.tim.2005.10.003. 16253506

[11] Khafipour E, Krause D, Plaizier J. 2009. A grain-based subacute ruminal acidosis challenge causes translocation of lipopolysaccharide and triggers inflammation. J Dairy Sci 92:1060–1070. doi:10.3168/jds.2008-1389. 19233799

[12] Eckel EF, Ametaj BN. 2020. Bacterial endotoxins and their role in periparturient diseases of dairy cows: mucosal vaccine perspectives. Dairy 1:61–90. doi:10.3390/dairy1010006.

[13] LaCount D, Drackley J, Weigel D. 1995. Responses of dairy cows during early lactation to ruminal or abomasal administration of L-carnitine. J Dairy Sci 78:1824–1836. doi:10.3168/jds.S0022-0302(95)76807-2. 8786266

[14] Arumugam M, Raes J, Pelletier E, Le Paslier D, Yamada T, Mende DR, Fernandes GR, Tap J, Bruls T, Batto JM, Bertalan M, Borruel N, Casellas F, Fernandez L, Gautier L, Hansen T, Hattori M, Hayashi T, Kleerebezem M, Kurokawa K, Leclerc M, Levenez F, Manichanh C, Nielsen HB, Nielsen T, Pons N, Poulain J, Qin J, Sicheritz-Ponten T, Tims S, Torrents D, Ugarte E, Zoetendal EG, Wang J, Guarner F, Pedersen O, de Vos WM, Brunak S, Doré J, Antolín M, Artiguenave F, Blottiere HM, Almeida M, Brechot C, Cara C, Chervaux C, Cultrone A, Delorme C, Denariaz G, Dervyn R, Foerstner KU, Friss C, van de Guchte M, Guedon E, Haimet F, Huber W, van Hylckama-Vlieg J, Jamet A, Juste C, Kaci G, Knol J, Lakhdari O, Layec S, Le Roux K, Maguin E, Mérieux A, Melo Minardi R, M'rini C, Muller J, Oozeer R, Parkhill J, Renault P, Rescigno M, Sanchez N, Sunagawa S, Torrejon A, Turner K, Vandemeulebrouck G, Varela E, Winogradsky Y, Zeller G, Weissenbach J, Ehrlich SD, Bork P, MetaHIT Consortium. 2011. Enterotypes of the human gut microbiome. Nature 473:174–180. doi:10.1038/nature09944. 21508958 PMC3728647

[15] Kononov SU, Meyer J, Frahm J, Kersten S, Kluess J, Meyer U, Huber K, Dänicke S. 2020. Effects of dietary L-carnitine supplementation on platelets and erythrogram of dairy cows with special emphasis on parturition. Dairy 2:1–13. doi:10.3390/dairy2010001.

[16] Meyer J, Daniels SU, Grindler S, Tröscher-Mußotter J, Alaedin M, Frahm J, Hüther L, Kluess J, Kersten S, von Soosten D, Meyer U, Most E, Eder K, Sauerwein H, Seifert J, Huber K, Rehage J, Dänicke S. 2020. Effects of a dietary L-carnitine supplementation on performance, energy metabolism and recovery from calving in dairy cows. Animals 10:342. doi:10.3390/ani10020342. PMC7070952 32098123

[17] Meyer J, Kononov SU, Grindler S, Tröscher-Mußotter J, Alaedin MT, Frahm J, Hüther L, Kluess J, Kersten S, von Soosten D, Meyer U, Most E, Eder K, Sauerwein H, Seifert J, Huber K, Wegerich A, Rehage J, Dänicke S. 2021. Dietary l-carnitine supplementation modifies the lipopolysaccharide-induced acute phase reaction in dairy cows. Animals 11:136. doi:10.3390/ani11010136. PMC7828073 33435209

[18] Hildebrand F, Nguyen TLA, Brinkman B, Yunta RG, Cauwe B, Vandenabeele P, Liston A, Raes J. 2013. Inflammation-associated enterotypes, host genotype, cage and inter-individual effects drive gut microbiota variation in common laboratory mice. Genome Biol 14:R4. doi:10.1186/gb-2013-14-1-r4. 23347395 PMC4053703

[19] Knights D, Ward TL, McKinlay CE, Miller H, Gonzalez A, McDonald D, Knight R. 2014. Rethinking “enterotypes.” Cell Host Microbe 16:433–437. doi:10.1016/j.chom.2014.09.013. 25299329 PMC5558460

[20] Lozupone CA, Stombaugh JI, Gordon JI, Jansson JK, Knight R. 2012. Diversity, stability and resilience of the human gut microbiota. Nature 489:220–230. doi:10.1038/nature11550. 22972295 PMC3577372

[21] Clemmons BA, Martino C, Schneider LG, Lefler J, Embree MM, Myer PR. 2019. Temporal stability of the ruminal bacterial communities in beef steers. Sci Rep 9:1–8. doi:10.1038/s41598-019-45995-2. 31266992 PMC6606625

[22] Coyte KZ, Schluter J, Foster KR. 2015. The ecology of the microbiome: networks, competition, and stability. Science 350:663–666. doi:10.1126/science.aad2602. 26542567

[23] Voolstra CR, Ziegler M. 2020. Adapting with microbial help: microbiome flexibility facilitates rapid responses to environmental change. BioEssays 42:2000004. doi:10.1002/bies.202000004. 32548850

[24] Welch CB, Lourenco JM, Davis DB, Krause TR, Carmichael MN, Rothrock MJ, Pringle TD, Callaway TR. 2020. The impact of feed efficiency selection on the ruminal, cecal, and fecal microbiomes of Angus steers from a commercial feedlot. J Anim Sci 98:skaa230. doi:10.1093/jas/skaa230. 32687166 PMC7392532

[25] Van Vliet P, Reijs J, Bloem J, Dijkstra J, De Goede R. 2007. Effects of cow diet on the microbial community and organic matter and nitrogen content of feces. J Dairy Sci 90:5146–5158. doi:10.3168/jds.2007-0065. 17954755

[26] Krautkramer KA, Fan J, Bäckhed F. 2020. Gut microbial metabolites as multi-kingdom intermediates. Nat Rev Microbiol 19:77–94. doi:10.1038/s41579-020-0438-4. 32968241

[27] Litvak Y, Byndloss MX, Bäumler AJ. 2018. Colonocyte metabolism shapes the gut microbiota. Science 362:eaat9076. doi:10.1126/science.aat9076. 30498100 PMC6296223

[28] Fransen F, van Beek AA, Borghuis T, Aidy SE, Hugenholtz F, van der Gaast-de Jongh C, Savelkoul HFJ, De Jonge MI, Boekschoten MV, Smidt H, Faas MM, de Vos P. 2017. Aged gut microbiota contributes to systemical inflammaging after transfer to germ-free mice. Front Immunol 8:1385. doi:10.3389/fimmu.2017.01385. 29163474 PMC5674680

[29] Liu J, Xu T, Zhu W, Mao S. 2014. High-grain feeding alters caecal bacterial microbiota composition and fermentation and results in caecal mucosal injury in goats. Br J Nutr 112:416–427. doi:10.1017/S0007114514000993. 24846282

[30] Huang S, Ji S, Yan H, Hao Y, Zhang J, Wang Y, Cao Z, Li S. 2020. The day‐to‐day stability of the ruminal and fecal microbiota in lactating dairy cows. Microbiologyopen 9:e990. doi:10.1002/mbo3.990. 32175695 PMC7221419

[31] Oliphant K, Allen-Vercoe E. 2019. Macronutrient metabolism by the human gut microbiome: major fermentation by-products and their impact on host health. Microbiome 7:1–15. doi:10.1186/s40168-019-0704-8. 31196177 PMC6567490

[32] Kalantar-Zadeh K, Berean KJ, Burgell RE, Muir JG, Gibson PR. 2019. Intestinal gases: influence on gut disorders and the role of dietary manipulations. Nat Rev Gastroenterol Hepatol 16:733–747. doi:10.1038/s41575-019-0193-z. 31520080

[33] Singhal R, Shah YM. 2020. Oxygen battle in the gut: hypoxia and hypoxia-inducible factors in metabolic and inflammatory responses in the intestine. J Biol Chem 295:10493–10505. doi:10.1074/jbc.REV120.011188. 32503843 PMC7383395

[34] Weber GJ, van Sambeek DM, Pearce SC. 2020. Hypoxia and hypercapnia diminish intestinal barrier integrity in a human enteroid model system. FASEB j 34:1–1. doi:10.1096/fasebj.2020.34.s1.05850.

[35] Allen MS, Piantoni P. 2013. Metabolic control of feed intake: implications for metabolic disease of fresh cows. Vet Clin North Am Food Anim Pract 29:279–297. doi:10.1016/j.cvfa.2013.04.001. 23809892

[36] Gianesella M, Morgante M, Cannizzo C, Stefani A, Dalvit P, Messina V, Giudice E. 2010. Subacute ruminal acidosis and evaluation of blood gas analysis in dairy cow. Vet Med Int 2010:392371. doi:10.4061/2010/392371. 20953375 PMC2952916

[37] Maas MN, Hintzen JCJ, Porzberg MRB, Mecinović J. 2020. Trimethyllysine: from carnitine biosynthesis to epigenetics. Int J Mol Sci 21:9451. doi:10.3390/ijms21249451. PMC7764450 33322546

[38] Skagen K, Trøseid M, Ueland T, Holm S, Abbas A, Gregersen I, Kummen M, Bjerkeli V, Reier-Nilsen F, Russell D, Svardal A, Karlsen TH, Aukrust P, Berge RK, Hov JER, Halvorsen B, Skjelland M. 2016. The carnitine-butyrobetaine-trimethylamine-N-oxide pathway and its association with cardiovascular mortality in patients with carotid atherosclerosis. Atherosclerosis 247:64–69. doi:10.1016/j.atherosclerosis.2016.01.033. 26868510

[39] Oikonomou G, Teixeira AGV, Foditsch C, Bicalho ML, Machado VS, Bicalho RC. 2013. Fecal microbial diversity in pre-weaned dairy calves as described by pyrosequencing of metagenomic 16S rDNA. Associations of *Faecalibacterium* species with health and growth. PLoS One 8:e63157. doi:10.1371/journal.pone.0063157. 23646192 PMC3639981

[40] Amin N, Schwarzkopf S, Kinoshita A, Tröscher-Mußotter J, Dänicke S, Camarinha-Silva A, Huber K, Frahm J, Seifert J. 2021. Evolution of rumen and oral microbiota in calves is influenced by age and time of weaning. Anim Microbiome 3:31. doi:10.1186/s42523-021-00095-3. 33883031 PMC8059317

[41] Kordy K, Gaufin T, Mwangi M, Li F, Cerini C, Lee DJ, Adisetiyo H, Woodward C, Pannaraj PS, Tobin NH, Aldrovandi GM. 2020. Contributions to human breast milk microbiome and enteromammary transfer of Bifidobacterium breve. PLoS One 15:e0219633. doi:10.1371/journal.pone.0219633. 31990909 PMC6986747

[42] Young W, Hine BC, Wallace OA, Callaghan M, Bibiloni R. 2015. Transfer of intestinal bacterial components to mammary secretions in the cow. PeerJ 3:e888. doi:10.7717/peerj.888. 25922791 PMC4411484

[43] Mohan R, Koebnick C, Schildt J, Mueller M, Radke M, Blaut M. 2008. Effects of *Bifidobacterium lactis* Bb12 supplementation on body weight, fecal pH, acetate, lactate, calprotectin, and IgA in preterm infants. Pediatr Res 64:418–422. doi:10.1203/PDR.0b013e318181b7fa. 18552710

[44] Korzhenkov AA, Tepliuk AV, Sidoruk KV, Voyushin KE, Patrushev MV, Kublanov IV, Toshchakov SV. 2021. A dataset of four probiotic *Bifidobacterium* strains genome assemblies. Data Brief 34:106710. doi:10.1016/j.dib.2020.106710. 33490330 PMC7807135

[45] Kim HS, Whon TW, Sung H, Jeong Y-S, Jung ES, Shin N-R, Hyun D-W, Kim PS, Lee J-Y, Lee CH. 2021. Longitudinal evaluation of fecal microbiota transplantation for ameliorating calf diarrhea and improving growth performance. Nat Commun 12:161. doi:10.1038/s41467-020-20389-5. 33420064 PMC7794225

[46] Malmuthuge N, Chen Y, Liang G, Goonewardene LA, Guan LL. 2015. Heat-treated colostrum feeding promotes beneficial bacteria colonization in the small intestine of neonatal calves. J Dairy Sci 98:8044–8053. doi:10.3168/jds.2015-9607. 26342981

[47] Santacruz A, Collado MC, García-Valdés L, Segura MT, Martín-Lagos JA, Anjos T, Martí-Romero M, Lopez RM, Florido J, Campoy C, Sanz Y. 2010. Gut microbiota composition is associated with body weight, weight gain and biochemical parameters in pregnant women. Br J Nutr 104:83–92. doi:10.1017/S0007114510000176. 20205964

[48] Christensen L, Roager HM, Astrup A, Hjorth MF. 2018. Microbial enterotypes in personalized nutrition and obesity management. Am J Clin Nutr 108:645–651. doi:10.1093/ajcn/nqy175. 30239555

[49] Fischer A, Delagarde R, Faverdin P. 2018. Identification of biological traits associated with differences in residual energy intake among lactating Holstein cows. J Dairy Sci 101:4193–4211. doi:10.3168/jds.2017-12636. 29398023

[50] Patil RD, Ellison MJ, Wolff SM, Shearer C, Wright AM, Cockrum RR, Austin KJ, Lamberson WR, Cammack KM, Conant GC. 2018. Poor feed efficiency in sheep is associated with several structural abnormalities in the community metabolic network of their ruminal microbes. J Anim Sci 96:2113–2124. doi:10.1093/jas/sky096. 29788417 PMC6095279

[51] Shabat SKB, Sasson G, Doron-Faigenboim A, Durman T, Yaacoby S, Miller MEB, White BA, Shterzer N, Mizrahi I. 2016. Specific microbiome-dependent mechanisms underlie the energy harvest efficiency of ruminants. ISME J 10:2958–2972. doi:10.1038/ismej.2016.62. 27152936 PMC5148187

[52] Uzan-Yulzari A, Turta O, Belogolovski A, Ziv O, Kunz C, Perschbacher S, Neuman H, Pasolli E, Oz A, Ben-Amram H. 2021. Neonatal antibiotic exposure impairs child growth during the first six years of life by perturbing intestinal microbial colonization. Nat Commun 12:443. doi:10.1038/s41467-020-20495-4. 33500411 PMC7838415

[53] Korpela K, Salonen A, Saxen H, Nikkonen A, Peltola V, Jaakkola T, de Vos W, Kolho K-L. 2020. Antibiotics in early life associate with specific gut microbiota signatures in a prospective longitudinal infant cohort. Pediatr Res 88:438–436. doi:10.1038/s41390-020-0761-5. 31954376

[54] Gonzalez-Recio O, Zubiria I, García-Rodríguez A, Hurtado A, Atxaerandio R. 2018. Signs of host genetic regulation in the microbiome composition in 2 dairy breeds: Holstein and Brown Swiss. J Dairy Sci 101:2285–2292. doi:10.3168/jds.2017-13179. 29274973

[55] Burbach K, Seifert J, Pieper DH, Camarinha‐Silva A. 2016. Evaluation of DNA extraction kits and phylogenetic diversity of the porcine gastrointestinal tract based on Illumina sequencing of two hypervariable regions. Microbiologyopen 5:70–82. doi:10.1002/mbo3.312. 26541370 PMC4767427

[56] Kaewtapee C, Burbach K, Tomforde G, Hartinger T, Camarinha-Silva A, Heinritz S, Seifert J, Wiltafsky M, Mosenthin R, Rosenfelder-Kuon P. 2017. Effect of *Bacillus subtilis* and *Bacillus licheniformis* supplementation in diets with low- and high-protein content on ileal crude protein and amino acid digestibility and intestinal microbiota composition of growing pigs. J Anim Sci Biotechnol 8:1–15. doi:10.1186/s40104-017-0168-2. 28469845 PMC5410705

[57] Bolyen E, Rideout JR, Dillon MR, Bokulich NA, Abnet CC, Al-Ghalith GA, Alexander H, Alm EJ, Arumugam M, Asnicar F, Bai Y, Bisanz JE, Bittinger K, Brejnrod A, Brislawn CJ, Brown CT, Callahan BJ, Caraballo-Rodríguez AM, Chase J, Cope EK, Da Silva R, Diener C, Dorrestein PC, Douglas GM, Durall DM, Duvallet C, Edwardson CF, Ernst M, Estaki M, Fouquier J, Gauglitz JM, Gibbons SM, Gibson DL, Gonzalez A, Gorlick K, Guo J, Hillmann B, Holmes S, Holste H, Huttenhower C, Huttley GA, Janssen S, Jarmusch AK, Jiang L, Kaehler BD, Kang KB, Keefe CR, Keim P, Kelley ST, Knights D, et al. 2019. Reproducible, interactive, scalable and extensible microbiome data science using QIIME 2. Nat Biotechnol 37:852–857. doi:10.1038/s41587-019-0209-9. 31341288 PMC7015180

[58] Callahan BJ, McMurdie PJ, Rosen MJ, Han AW, Johnson AJA, Holmes SP. 2016. DADA2: high-resolution sample inference from Illumina amplicon data. Nat Methods 13:581–583. doi:10.1038/nmeth.3869. 27214047 PMC4927377

[59] Pedregosa F, Varoquaux G, Gramfort A, Michel V, Thirion B, Grisel O, Blondel M, Prettenhofer P, Weiss R, Dubourg V. 2011. Scikit-learn: machine learning in Python. J Mach Learn Res 12:2825–2830.

[60] Quast C, Pruesse E, Yilmaz P, Gerken J, Schweer T, Yarza P, Peplies J, Glöckner FO. 2013. The SILVA ribosomal RNA gene database project: improved data processing and web-based tools. Nucleic Acids Res 41:D590–D596. doi:10.1093/nar/gks1219. 23193283 PMC3531112

[61] Cole JR, Wang Q, Fish JA, Chai B, McGarrell DM, Sun Y, Brown CT, Porras-Alfaro A, Kuske CR, Tiedje JM. 2014. Ribosomal Database Project: data and tools for high throughput rRNA analysis. Nucleic Acids Res 42:D633–D642. doi:10.1093/nar/gkt1244. 24288368 PMC3965039

[62] Wang Q, Garrity GM, Tiedje JM, Cole JR. 2007. Naive Bayesian classifier for rapid assignment of rRNA sequences into the new bacterial taxonomy. Appl Environ Microbiol 73:5261–5267. doi:10.1128/AEM.00062-07. 17586664 PMC1950982

[63] Yarza P, Yilmaz P, Pruesse E, Glöckner FO, Ludwig W, Schleifer K-H, Whitman WB, Euzéby J, Amann R, Rosselló-Móra R. 2014. Uniting the classification of cultured and uncultured bacteria and archaea using 16S rRNA gene sequences. Nat Rev Microbiol 12:635–645. doi:10.1038/nrmicro3330. 25118885

[64] Wickham H, Averick M, Bryan J, Chang W, McGowan L, François R, Grolemund G, Hayes A, Henry L, Hester J, Kuhn M, Pedersen T, Miller E, Bache S, Müller K, Ooms J, Robinson D, Seidel D, Spinu V, Takahashi K, Vaughan D, Wilke C, Woo K, Yutani H. 2019. Welcome to the Tidyverse. J Open Source Softw 4:1686. doi:10.21105/joss.01686.

[65] Walesiak M, Dudek A. 2020. The choice of variable normalization method in cluster analysis, p 325–340. *In* Soliman KS (ed), Education excellence and innovation management: a 2025 vision to sustain economic development during global challenges. International Business Information Management Association, Seville, Spain.

[66] Maechler M, Rousseeuw P, Struyf A, Hubert M, Hornik K, Studer M, Roudier P. 2014. Package ‘cluster’. https://mran.microsoft.com/snapshot/2014-12-11/web/packages/cluster/cluster.pdf.

[67] Kassambara A, Mundt F. 2017. Package ‘factoextra’: extract and visualize the results of multivariate data analyses. https://rpkgs.datanovia.com/factoextra/index.html.

[68] Thioulouse J, Dray S, Dufour A-B, Siberchicot A, Jombart T, Pavoine S. 2018. Multivariate analysis of ecological data with ade4. Springer-Verlag, New York, NY.

[69] Lengowski MB, Witzig M, Möhring J, Seyfang GM, Rodehutscord M. 2016. Effects of corn silage and grass silage in ruminant rations on diurnal changes of microbial populations in the rumen of dairy cows. Anaerobe 42:6–16. doi:10.1016/j.anaerobe.2016.07.004. 27451293

[70] Lee C, Lee S, Shin SG, Hwang S. 2008. Real-time PCR determination of rRNA gene copy number: absolute and relative quantification assays with *Escherichia coli*. Appl Microbiol Biotechnol 78:371–376. doi:10.1007/s00253-007-1300-6. 18074129

[71] Monteagudo-Mera A, Arthur J, Jobin C, Keku T, Bruno-Barcena J, Azcarate-Peril M. 2016. High purity galacto-oligosaccharides (GOS) enhance specific *Bifidobacterium* species and their metabolic activity in the mouse gut microbiome. Benef Microbes 7:247–264. doi:10.3920/BM2015.0114. 26839072 PMC4974821

[72] Wilkinson TJ, Huws SA, Edwards JE, Kingston-Smith AH, Siu-Ting K, Hughes M, Rubino F, Friedersdorff M, Creevey CJ. 2018. CowPI: a rumen microbiome focussed version of the PICRUSt functional inference software. Front Microbiol 9:1095. doi:10.3389/fmicb.2018.01095. 29887853 PMC5981159

[73] Wischer G, Boguhn J, Steingaß H, Schollenberger M, Hartung K, Rodehutscord M. 2013. Effect of monensin on in vitro fermentation of silages and microbial protein synthesis. Arch Anim Nutr 67:219–234. doi:10.1080/1745039X.2013.793050. 23679006

[74] Kenéz Á, Dänicke S, Rolle-Kampczyk U, von Bergen M, Huber K. 2016. A metabolomics approach to characterize phenotypes of metabolic transition from late pregnancy to early lactation in dairy cows. Metabolomics 12:1–11. doi:10.1007/s11306-016-1112-8.

[75] Bray JR, Curtis JT. 1957. An ordination of the upland forest communities of southern Wisconsin. Ecol Monogr 27:325–349. doi:10.2307/1942268.

[76] Clarke KR, Gorley R, Somerfield P, Warwick R. 2014. Change in marine communities: an approach to statistical analysis and interpretation. Primer-E Ltd., Devon, United Kingdom.

[77] Bates D, Mächler M, Bolker B, Walker S. 2014. Fitting linear mixed-effects models using lme4. arXiv 1406.5823 [stat.CO].

[78] Kuznetsova A, Brockhoff PB, Christensen RH. 2017. lmerTest package: tests in linear mixed effects models. J Stat Softw 82:1–26.

