# Supplementary figures and images for: Microbiome Clusters Disclose Physiologic Variances in Dairy Cows Challenged by Calving and Lipopolysaccharides
Source: mSystems. 2021 Oct 19;6(5):e00856-21. doi: 10.1128/mSystems.00856-21 (PMC8525563; doi:10.1128/mSystems.00856-21)

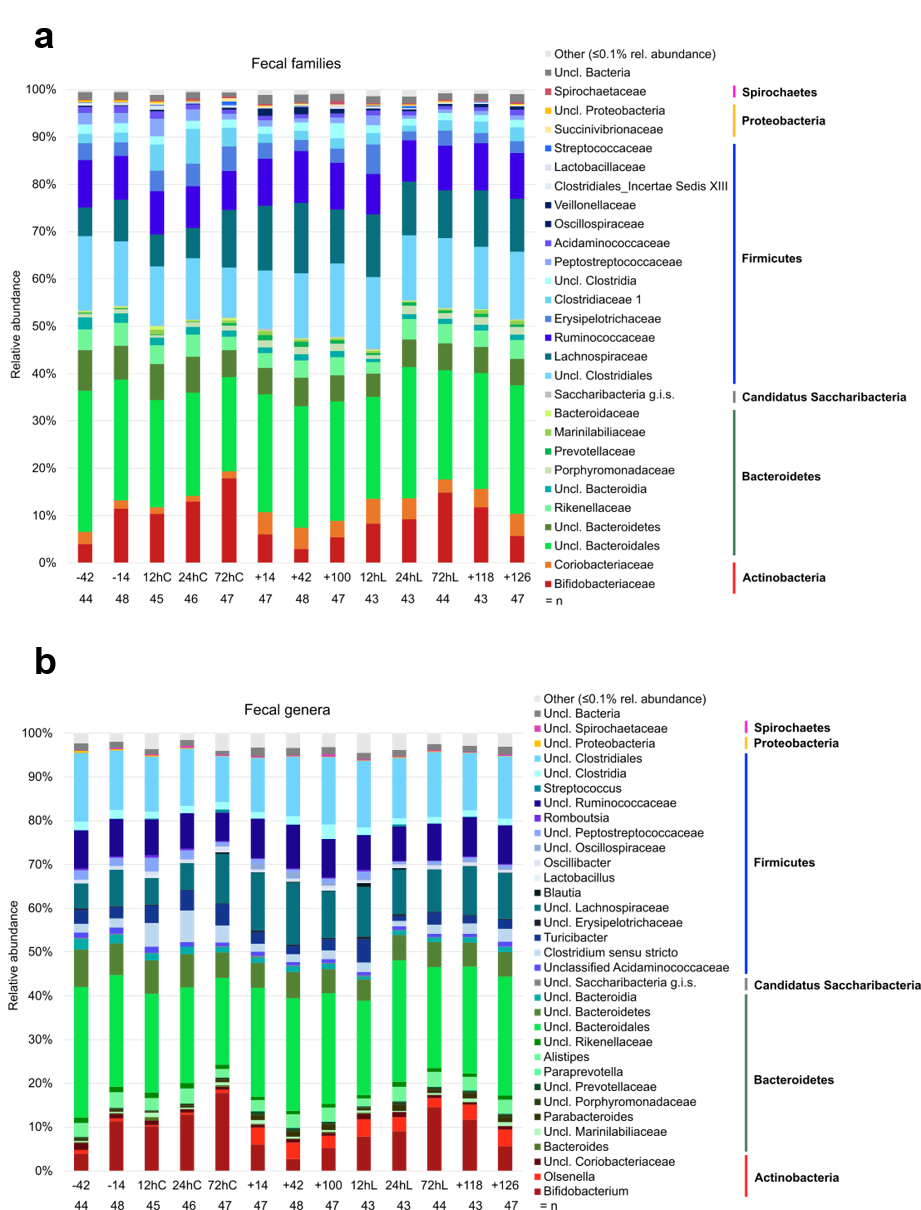

Supplement: FIG S1 [file msystems.00856-21-sf001.tif]

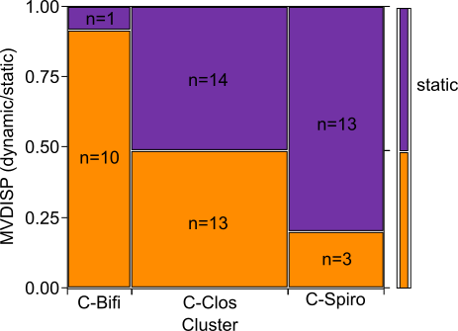

Supplement: FIG S2 [file msystems.00856-21-sf002.tif]

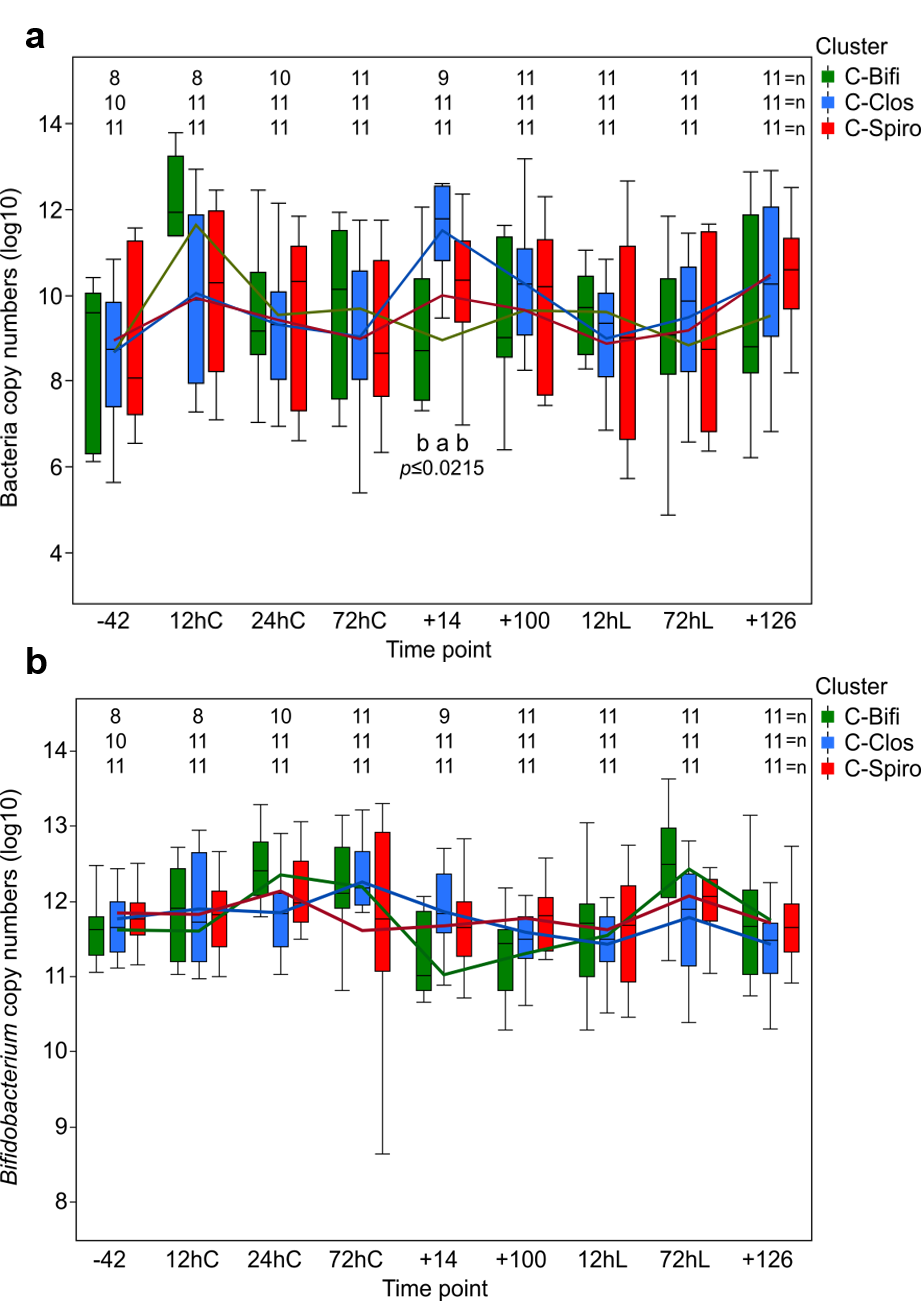

Supplement: FIG S3 [file msystems.00856-21-sf003.tif]

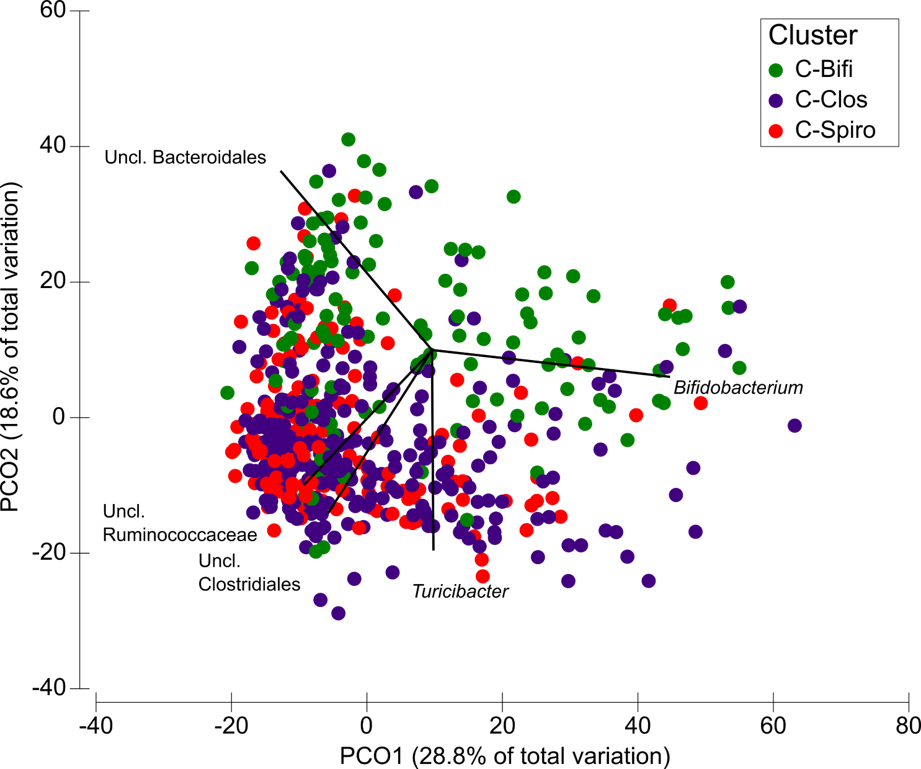

Supplement: FIG S4 [file msystems.00856-21-sf004.tif]

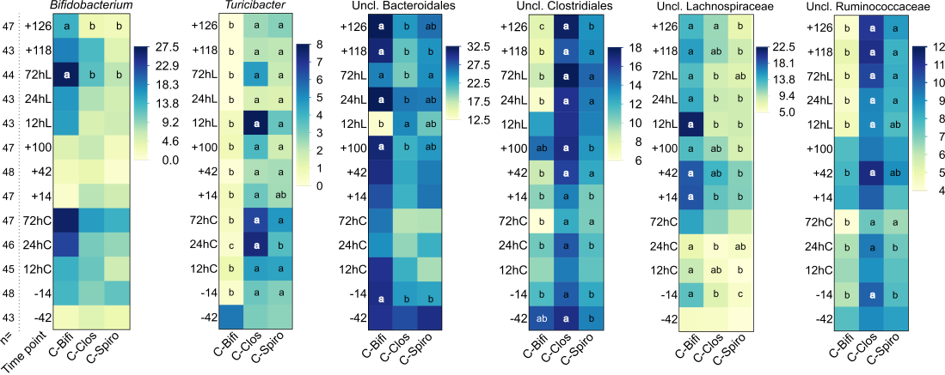

Supplement: FIG S5 [file msystems.00856-21-sf005.tif]

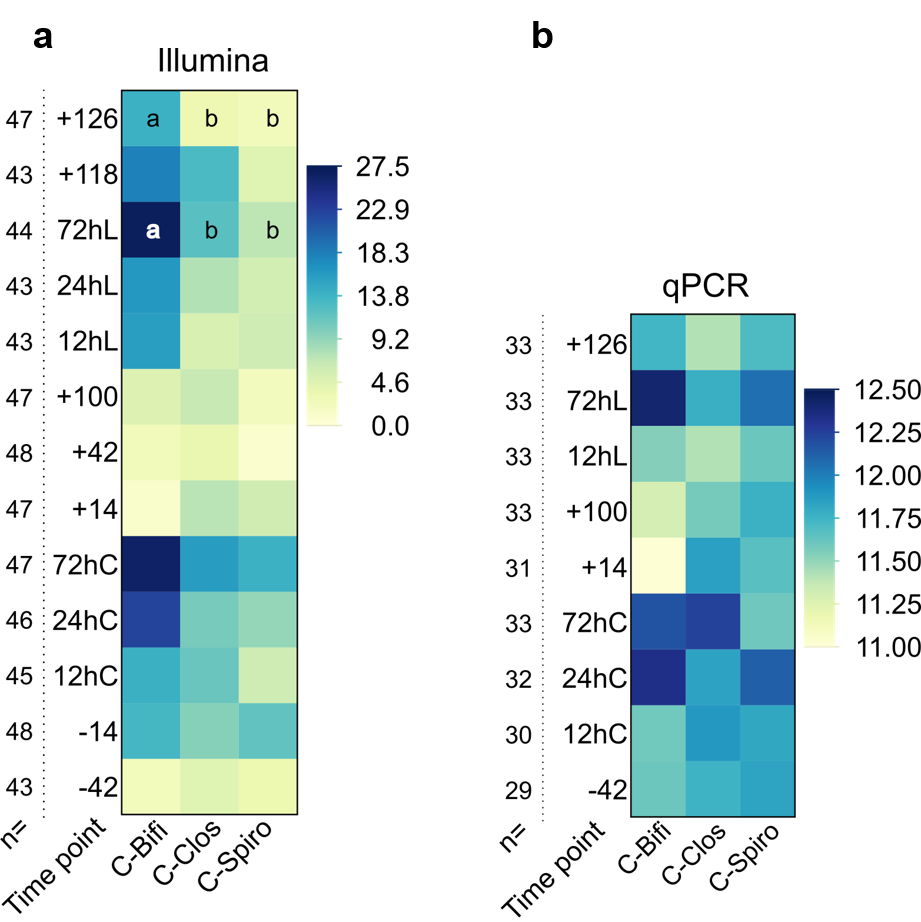

Supplement: FIG S6 [file msystems.00856-21-sf006.tif]

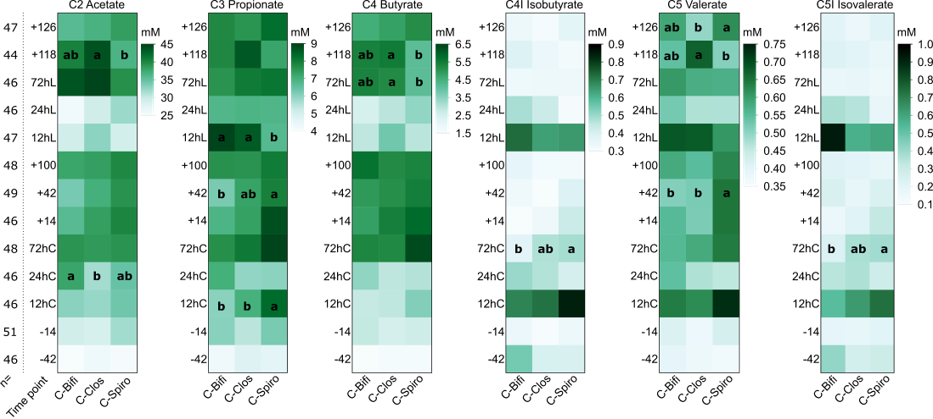

Supplement: FIG S7 [file msystems.00856-21-sf007.tif]

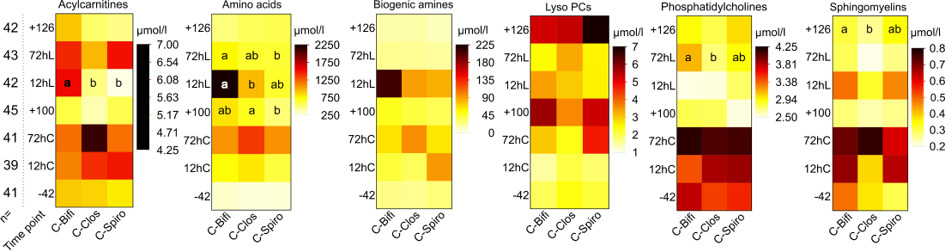

Supplement: FIG S8 [file msystems.00856-21-sf008.tif]

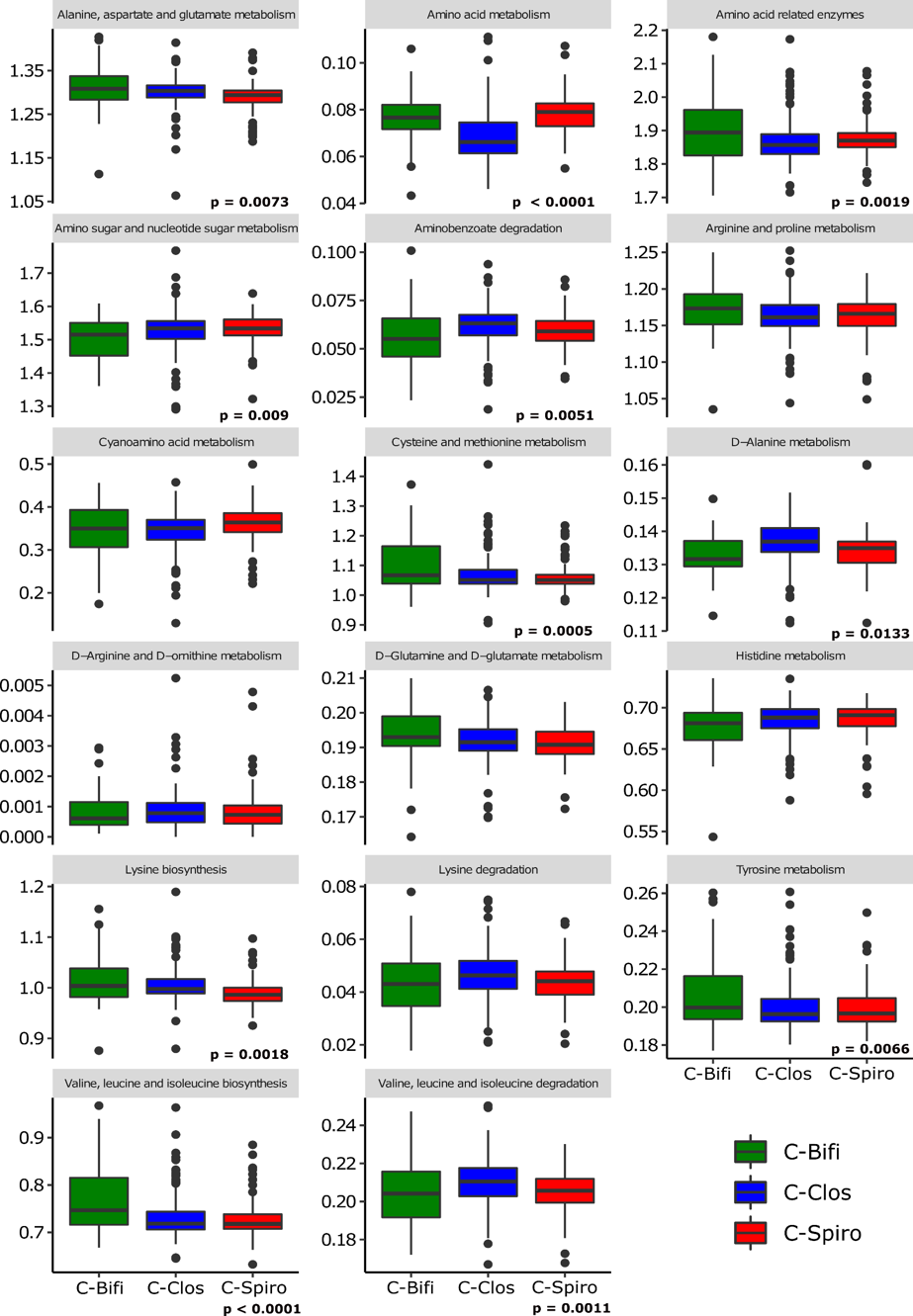

Supplement: FIG S9 [file msystems.00856-21-sf009.tif]

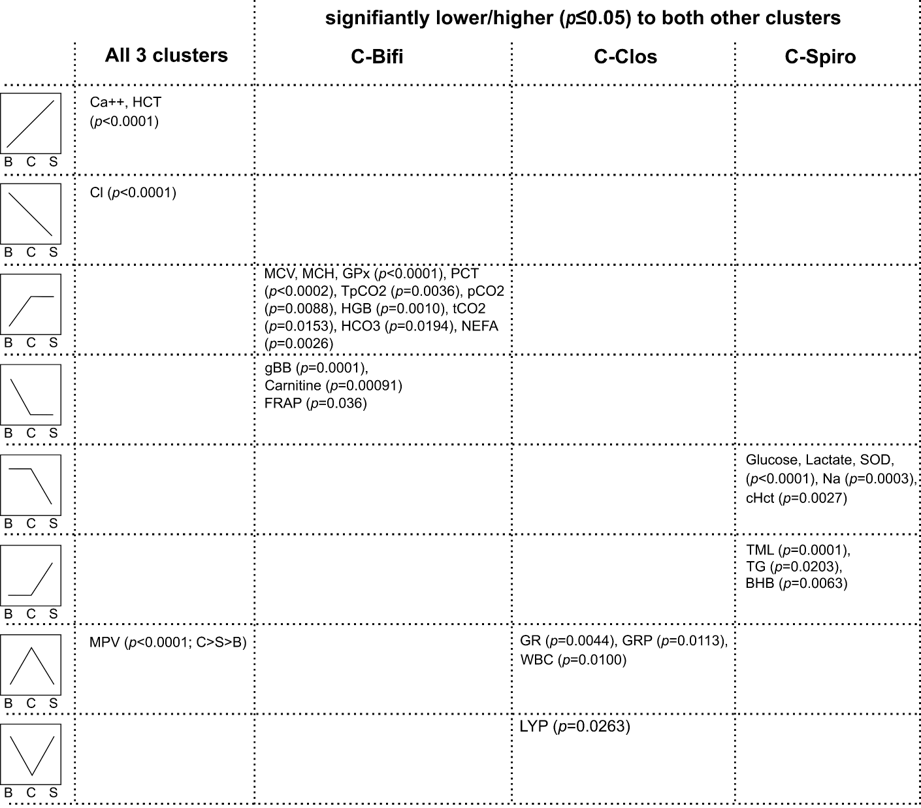

Supplement: FIG S10 [file msystems.00856-21-sf010.tif]
